# Is Adolescent E-Cigarette Use Associated With Subsequent Smoking? A New Look

**DOI:** 10.1093/ntr/ntab243

**Published:** 2021-11-20

**Authors:** Ruoyan Sun, David Mendez, Kenneth E Warner

**Affiliations:** 1 Department of Health Care Organization and Policy, School of Public Health, University of Alabama at Birmingham, Birmingham, AL, USA; 2 Department of Health Management and Policy, School of Public Health, University of Michigan, Ann Arbor, MI, USA

## Abstract

**Introduction:**

Prospective studies have consistently reported a strong association between e-cigarette use and subsequent cigarette smoking, but many failed to adjust for important risk factors.

**Methods:**

Using longitudinal data from the Population Assessment of Tobacco and Health (PATH) Study, we employed multivariable logistic regressions to assess the adolescent vaping-to-smoking relationship, with four regressions (Models 1–4) sequentially adding more risk factors.

Our sample included all waves (waves 1–5) of the PATH Study.

**Results:**

The association between ever e-cigarette use and subsequent cigarette smoking decreased substantially in magnitude when adding more control variables, including respondents’ sociodemographic characteristics, exposure to tobacco users, cigarette susceptibility, and behavioral risk factors. Using the most recent data (waves 4–4.5 and waves 4.5–5), this association was not significant in the most complete model (Model 4). Using wave 4.5–5 data, the adjusted odds ratio (aOR) for ever e-cigarette use at initial wave and subsequent past 12-month smoking declined from 4.07 (95% confidence interval [CI, 2.86−5.81) in Model 1, adjusting only for sociodemographic characteristics, to 1.35 (95% CI, 0.84−2.16) in Model 4, adjusting for all potential risk factors. Similarly, the aOR of ever e-cigarette use and past 30-day smoking at wave 5 decreased from 3.26 (95% CI, 1.81−5.86) in Model 1 to 1.21 (95% CI, 0.59−2.48) with all covariates (Model 4).

**Conclusions:**

Among adolescent never cigarette smokers, those who had ever used e-cigarettes at baseline, compared with never e-cigarette users, exhibited modest or non-significant increases in subsequent past 12-month or past 30-day smoking when adjusting for behavioral risk factors.

## Introduction

Cigarette smoking among US adolescents has steadily decreased over the last quarter century. In 2020, 4.6% of high school and 1.6% of middle school students reported having smoked in the past 30 days.^1^ However, while smoking has declined, in recent years electronic cigarettes have become popular. In 2019, 27.5% of high school and 10.5% of middle school students used e-cigarettes (vaped) in the past 30 days.^2^ Although these rates dropped substantially in 2020 and 2021, e-cigarettes remain the most popular nicotine or tobacco product among US adolescents.^1,3^

One concern is that e-cigarette use may lead young people to try cigarettes when, absent the experience of vaping, they would not have done so. Studies have found strong associations between youth and young adult vaping and subsequent cigarette smoking.^4–22^ In a meta-analysis of nine US studies, Soneji et al.^23^ reported that the pooled odds ratio for subsequent use of cigarettes among previously non-smoking vapers, compared to youth who had never vaped or smoked, was 3.50 (95% CI, 2.38−5.16). The ratio was 4.28 (95% CI, 2.52−7.27) for “current” use of cigarettes (having smoked in the past 30 days).^22^ A systematic review by Khouja et al.,^24^ covering 17 studies of youth and young adults, found strong evidence for a significant association between e-cigarette use and later smoking with an odds ratio of 4.59 (95% CI, 3.60−5.85).

An essential question, prompted by these findings, is whether these associations reflect a causal relationship, which these studies per se cannot determine. There are two competing views of the relationship. The gateway hypothesis posits that the use of e-cigarettes causes the subsequent use of conventional cigarettes.^25–27^ In contrast, the common liability theory suggests that youth who vape and subsequently smoke would have tried cigarettes in the absence of vaping, since the two behaviors reflect a common propensity for risky behaviors.^28–30^ Note that none of the authors of the prospective studies explicitly claim that their study supports either theory about the relationship between vaping and subsequent cigarette smoking.^6–22^ Indeed, the vast majority do not even mention the competing views. However, others have interpreted these findings as implying support for the gateway theory. For example, referring to the Khouja et al. review,^24^ a prominent anti-vaping scholar/activist wrote a blog post entitled “Convincing analysis that e-cigs are a gateway to cigarette smoking from studies around the world.” ^31^ However, Khouja et al. concluded “[F]indings from published studies do not provide clear evidence that this is explained by a gateway effect rather than shared common causes of both e-cigarette use and smoking.” ^24^

Proponents of the common liability theory assert that the prospective studies have failed to account adequately for factors that reflect this common liability.^28–30,32–34^ These could include peer and familial influences, use of other psychoactive substances, such as other tobacco products, marijuana, and alcohol (indicative of risk-taking propensity), and indices of sensation seeking or rebelliousness. However, the two prospective studies that have covered the most categories of risk factors have found a robust positive and significant relationship.^12,21^ No previous study has reported the absence of a statistically significant increase in the odds of smoking associated with previous vaping.

All previous prospective studies adjusted for respondents’ basic demographics (sex, age, race/ethnicity).^6–22^ Many adjusted for socioeconomic status measures.^6–9,11,12,14,16–18,20–22^ Some also adjusted for psychosocial variables (such as sensation seeking, intentions to smoke, perceived norms, and rebelliousness),^9,12,14,16–22^ susceptibility to smoking,^6,8,10–13^ and exposure to tobacco users (friends’ smoking, family tobacco use, and secondhand smoke).^6,8–10,12–14,17,21^ A few adjusted for other tobacco use,^13,19,21^ and other non-tobacco drug use.^10,12,14–16,21^ Reviewing the evidence, a committee of the National Academies of Science, Engineering and Medicine “considered studies that adjusted for a more comprehensive set of covariates as stronger evidence.” ^26^

No previous study has included all of the covariates in the present study. The two studies that incorporated the most variables similar to those in the present study, Leventhal et al.^12^ and Watkins et al.,^21^ found lower than average adjusted odds ratios (aORs) for the vaping-to-smoking relationship. However, Leventhal et al. did not consider the use of other tobacco products,^12^ while Watkins et al. did not include cigarette susceptibility or marijuana use in their main analysis^21^ (see Supplement eTable 1 for details on the individual studies, including which categories of independent variables they included).

In this study, we analyze longitudinal data from all waves of the Population Assessment of Tobacco and Health (PATH) Study (wave 1: 2013–2014; wave 2: 2014–2015; wave 3: 2015–2016; wave 4: 2016–2017; wave 4.5: 2017–2018; and wave 5: 2018–2019). We add to the literature by explicitly, and sequentially, examining how the inclusion of potential risk factors affects the magnitude and statistical significance of the vaping-to-smoking relationship. As with all previous studies, we include respondents’ sociodemographic characteristics. Then, in sequence, we include additional control variables, each of which has been used in prior studies. With one exception,^21^ none of those previous studies has included representatives of each category of control variables that we employ: the adolescents’ exposure to tobacco users (family members who use tobacco, secondhand smoke exposure, friends who use tobacco); cigarette smoking susceptibility; and behavioral risk factors, specifically including respondents’ previous use of other tobacco products, alcohol, and marijuana as measures of adolescents’ proclivity for use of psychoactive substances. Only three previous studies have adjusted for use of other tobacco products.^13,19,21^ To the best of our knowledge, the present study is the first one to adjust simultaneously for cigarette susceptibility, other tobacco product use, and use of alcohol and marijuana.

As with the previous studies, ours is not intended to resolve whether the vaping-to-smoking association represents a causal one (the gateway theory) or a coincidental one (the common liability theory). It is extremely challenging for studies using observational data to answer such questions. Rather, we explore whether inclusion of a more substantial set of covariates affects the finding of a statistically significant association between vaping at baseline and subsequent smoking. (We revisit the gateway-common liability issue in the Discussion section.)

## Methods

PATH is a nationally representative, longitudinal cohort study of US adults and youth aged 12 years and older. PATH employs a four-stage, stratified probability sample design to select adults and youth (12–17) from the US civilian, non-institutionalized population. Computer-assisted personal interviewing is used to collect self-reported data on tobacco use and related health behaviors. A full description of the PATH design and methods is available elsewhere.^35^ Our sample included youth who participated in consecutive waves of PATH, including waves 1–2 (2013–2015), 2–3 (2014–2016), 3–4 (2015–2017), 4–4.5 (2016–2018), and 4.5–5 (2017–2019). The weighted response rates for the wave 1 cohort in subsequent waves were 87.3%, 83.3%, 79.5%, 74.6%, and 72.3%, respectively. In wave 4, the survey added new respondents to replenish the sample. 40% of the wave 4 sample consisted of these new respondents. The weighted wave 4.5 and wave 5 response rates for the replenished wave 4 cohort were 89.1% and 83.5%.^36^ We study those youth who had never smoked cigarettes by the initial wave in each pair of waves.

### Measures

The principal independent variable is use of an e-cigarette at least once by the time of the survey in the initial wave in each pair of waves. We linked measures of sociodemographic characteristics, exposure to tobacco users, susceptibility to cigarette smoking, and behavioral risk factors of adolescent never cigarette smokers in the initial wave with their smoking behaviors in the subsequent wave one year later. We describe the specific covariates in each of these domains immediately below. The dependent variables, smoking behaviors at the subsequent wave, captured whether respondents had smoked at all during the past 12 months (even one puff on a cigarette) and whether they had smoked at all during the past 30 days (again, even one puff).

#### Sociodemographic Characteristics

Sociodemographic variables included age (12–14 vs. 15–17), sex (male vs. female), race/ethnicity (non-Hispanic white, non-Hispanic black, Hispanic, and non-Hispanic other), highest parental education (high school/GED or less, some college, college graduate or above), household income (<$50 000, $50 000–$100 000, >$100 000), and school grades (*≥* mostly B’s vs. not).

#### Exposure to Tobacco Users

Family tobacco use (0 vs. 1) was evaluated by asking if anyone living with the respondent uses cigarettes, smokeless tobacco, cigars, cigarillos, filtered cigars, or any other form of tobacco. Youth exposure to secondhand smoke (0 vs. 1) was determined by asking respondents if they had been around others who were smoking, including at home, in a car, at school, or outdoors, for at least a total of an hour during the past seven days. Friends’ tobacco use (0 vs. 1) was scored as 1 if participants provided a positive number to any of the questions asking, “How many of your best friends use [tobacco product]?” The tobacco products included in these questions are cigarettes, e-cigarettes, cigarillos, snus, and smokeless tobacco.

#### Susceptibility to Cigarette Smoking

Based on previous literature,^37,38^ we constructed a bivariate measure of cigarette susceptibility (0 vs. 1) from four survey questions. Youth never-smokers were asked, “Have you ever been curious about smoking a cigarette?”, “Do you think you will smoke a cigarette in the next year?”, “Do you think that you will try a cigarette soon?”, and “If one of your best friends were to offer you a cigarette, would you smoke it?” Participants answering “not at all curious” to the first question and “definitely not” to the last three were considered not susceptible to smoking; the rest were regarded as susceptible.

#### Behavioral Risk Factors

Behavioral risk factors include past 12-month use of alcohol (0 vs. 1) and marijuana (0 vs. 1) and ever use of other tobacco products (0 vs. 1), including cigar, pipe, hookah, snus, smokeless tobacco, bidi, kretek, and dissolvable tobacco.

### Analysis

All analyses were conducted using Stata version 16 (StataCorp LLC, College Station, TX). We performed multivariable logistic regressions with complete case analysis to examine the association between ever e-cigarette use at baseline and cigarette smoking a year later, adjusting for the previously identified variables. We incorporated baseline sample weights provided by PATH, using Fay’s variant of balanced repeated replication,^35^ to produce nationally representative results. These weights adjust for the potential impact of non-response.^36^

We performed four different models of multivariable logistic regressions. Model 1 adjusted only for sociodemographic variables. Model 2 added covariates measuring exposure to tobacco users. Model 3 added susceptibility to smoking cigarettes. Finally, Model 4 added ever use of other tobacco products, past 12-month use of alcohol, and past 12-month use of marijuana.

## Results

Table 1 shows the baseline characteristics of our most recent sample in waves 4.5–5, a total of 11 560 never-smokers. The weighted sample is approximately 50% male, and slightly more than half of the population were 12–14 years old, the rest 15–17. A majority were non-Hispanic white (51.4%). Parents’ education background was high school/GED or less (26.0%); some college (28.8%); and college graduate or higher (45.2%). 39.7% of the families made < $50 000 annually, with 26.5% between $50 000 and $100 000 and 33.8% >$100 000. 74.4% of respondents earned school grades *≥* mostly B’s. 27.6% of participants had at least one family member currently using tobacco products. 26.6% of participants were exposed to secondhand smoke in the past 7 days, and 32.2% had at least one best friend who used tobacco products. 26.1% of the sample were susceptible to cigarettes. 11.3% had ever vaped. 4.3% reported ever use of other tobacco products (other than cigarettes and e-cigarettes), 22.2% used alcohol in the past 12 months and 3.4% used marijuana. Baseline characteristics from other waves, largely similar, are presented in Supplement eTables 2A–D.

**Table 1.** Sample Characteristics Among Never Cigarette Smokers at Baseline, PATH Study Wave 4.5 (*N* = 11 560).

	Never cigarette smokers by wave 4.5	
	Proportion (%)	Weighted proportion (%, 95% CI)
Sociodemographic characteristics		
*Sex*		
Male	51.8	50.7 (50.3−51.1)
Female	48.2	49.3 (49.0−49.7)
*Age*		
12–14	50.7	53.9 (53.5−54.3)
15–17	49.3	46.1 (45.7−46.5)
*Race/ethnicity*		
Non-Hispanic white	46.2	51.4 (51.0−51.8)
Non-Hispanic black	13.4	13.5 (13.2−13.8)
Hispanic	30.6	24.4 (24.1−24.8)
Non-Hispanic other	9.8	10.7 (10.4−11.0)
*Highest parental education*		
High school/GED or less	29.4	26.0 (24.9−27.1)
Some college	29.7	28.8 (27.5−30.2)
College or higher	40.9	45.2 (43.7−46.7)
*Household income*		
<50k	44.0	39.7 (38.5−40.9)
50k to 100k	25.7	26.5 (25.4−27.6)
>100k	30.3	33.8 (32.4−35.4)
*Grades ≥ mostly B’s*		
Yes	72.9	74.4 (73.5−75.3)
No	27.1	25.6 (24.7−26.5)
**Exposure to tobacco users**		
*Family tobacco use*		
Yes	27.5	27.6 (26.5−28.8)
No	72.5	72.4 (71.2−73.6)
*Secondhand smoke*		
Yes	26.5	26.6 (25.6−27.6)
No	73.5	73.4 (72.4−74.4)
*Friends’ tobacco use*		
Yes	32.5	32.2 (31.1−33.3)
No	67.5	67.8 (66.7−68.9)
**Susceptibility**		
*Susceptible to cigarettes*		
Yes	26.4	26.1 (25.2−27.1)
No	73.6	73.9 (72.9−74.8)
**Behavioral risk factors**		
*Ever vaped*		
Yes	11.3	11.3 (10.6−11.9)
No	88.7	88.7 (88.1−89.4)
Ever used other tobacco products*		
Yes	4.4	4.3 (3.9−4.6)
No	95.6	95.7 (95.4−96.1)
*Used alcohol in past 12 months*		
Yes	21.9	22.2 (21.1−23.3)
No	78.2	77.8 (76.7−78.9)
*Used marijuana in past 12 months*		
Yes	3.5	3.4 (3.0−3.9)
No	96.5	96.6 (96.1−97.0)

*Other tobacco products include cigar, pipe, hookah, snus, smokeless tobacco, bidi, kretek, and dissolvable tobacco.

Table 2 presents weighted proportions of our baseline population by risk factors in wave 4.5 (excluding sociodemographic characteristics) who reported cigarette smoking in wave 5. Among never-smokers who had vaped by wave 4.5, 9.4% (CI 95%, 7.4−11.8) reported past 12-month cigarette use in wave 5, compared to 1.9% (CI 95%, 1.7−2.3) of never-vapers. Similarly, baseline participants who had vaped were significantly more likely to smoke in the past 30 days, 3.2% (CI 95%, 2.2−4.7) versus 0.7% (CI 95%, 0.5−0.9). Covariates measuring exposure to tobacco users (family tobacco use, secondhand smoke, and friends’ tobacco use), susceptibility to cigarette smoking and behavioral risk factors (other tobacco use, use of alcohol and marijuana) were all significantly associated with increased likelihood of smoking in wave 5. (See Supplement eTables 3A–D for the other waves.)

**Table 2.** Weighted Proportions of Baseline (Wave 4.5) Never Cigarette Smokers Using Cigarettes by Risk Factors (Excluding Sociodemographic Characteristics), PATH Study Wave 5 (*N* = 11 560)

Risk factors	Past 12-month cigarette smoking (%, 95% CI)	Past 30-day cigarette smoking (%, 95% CI)
Ever vaped*		
Yes	9.4 (7.4−11.8)	3.2 (2.2−4.7)
No	1.9 (1.7−2.3)	0.7 (0.5−0.9)
**Exposure to tobacco users**		
*Family tobacco use*		
Yes	4.8 (4.0−5.8)	1.8 (1.3−2.5)
No	1.8 (1.5−2.1)	0.6 (0.4−0.8)
*Secondhand smoke*		
Yes	5.1 (4.1−6.4)	1.9 (1.3−2.6)
No	1.7 (1.4−2.1)	0.6 (0.4−0.8)
*Friends’ tobacco use*		
Yes	5.8 (4.9−6.9)	3.2 (2.5−4.2)
No	1.3 (1.0−1.6)	0.5 (0.4−0.8)
**Susceptibility**		
*Susceptible to cigarettes*		
Yes	6.4 (5.4−7.6)	2.1 (1.5−2.8)
No	1.3 (1.0−1.6)	0.4 (0.3-−0.7)
**Behavioral risk factors**		
Ever used other tobacco products**		
Yes	13.7 (9.6−19.2)	4.2 (2.3−7.3)
No	2.2 (1.9−2.6)	0.8 (0.6−1.0)
*Used alcohol in past 12 months*		
Yes	5.9 (4.8−7.4)	2.1 (1.5−3.0)
No	1.8 (1.5−2.1)	0.6 (0.4−0.8)
*Used marijuana in past 12 months*		
Yes	10.3 (7.0−15.0)	4.2 (2.1−8.1)
No	2.1 (1.8−2.4)	0.7 (0.6−0.9)

* All risk factors are significant at *p* < .001 using Pearson’s chi-squared test of independence.

** Other tobacco products include cigar, pipe, hookah, snus, smokeless tobacco, bidi, kretek, and dissolvable tobacco.

In Table 3, we report aORs for the association between ever e-cigarette use by initial wave and subsequent past 12-month smoking. Across all four models, ever e-cigarette use is positively associated with subsequent cigarette smoking, but as we move from Model 1 to Model 4 the aOR becomes successively smaller across all waves and is non-significant (at the 5% level) in Model 4 for the two most recent wave comparisons, waves 4–4.5 and waves 4.5–5. For waves 1–2, the aOR decreases from 5.55 (95% CI, 3.87−7.97) in Model 1 to 2.09 (95% CI, 1.26−3.48) in Model 4; for waves 2–3, 5.93 (95% CI, 4.07−8.63) to 2.10 (95% CI, 1.33−3.30); for waves 3–4, 5.53 (95% CI, 4.11−7.44) to 2.25 (95% CI, 1.55−3.27); for waves 4–4.5, 4.96 (95% CI, 3.66−6.72) to 1.40 (95% CI, 0.91−2.14); and for waves 4.5–5, 4.07 (95% CI, 2.86−5.81) to 1.35 (95% CI, 0.84−2.16).

**Table 3.** Weighted Association of Ever E-Cig Use (Initial Wave) With Subsequent Past 12-Month Cigarette Use (Next Wave) Among US Youth in the PATH Study

	Reported subsequent past 12-month cigarette smoking			
Waves	Model 1 aOR (95% CI)	Model 2 aOR (95% CI)	Model 3 aOR (95% CI)	Model 4 aOR (95% CI)
Waves 1–2	**5.55** **(3.87**−**7.97)**	**4.98** **(3.38**−**7.34)**	**3.34** **(2.24**−**4.98)**	**2.09** **(1.26**−**3.48)**
	*p* < .001	*p* < .001	*p* < .001	*p* = .005
Waves 2–3	**5.93** **(4.07**−**8.63)**	**3.61** **(2.46**−**5.29)**	**2.95** **(2.03**−**4.28)**	**2.10** **(1.33**−**3.30)**
	*p* < .001	*p* < .001	*p* < .001	*p* = .002
Waves 3–4	**5.53** **(4.11**−**7.44)**	**3.77** **(2.80**−**5.07)**	**3.16** **(2.36**−**4.23)**	**2.25** **(1.55**−**3.27)**
	*p* < .001	*p* < .001	*p* < .001	*p* < .001
Waves 4–4.5	**4.96** **(3.66**−**6.72)**	**3.29** **(2.38**−**4.56)**	**2.71** **(1.99**−**3.68)**	1.40 (0.91−2.14)
	*p* < .001	*p* < .001	*p* < .001	*p* =.12
Waves 4.5–5	**4.07** **(2.86**−**5.81)**	**2.28** **(1.60**−**3.25)**	**1.92** **(1.34**−**2.75)**	1.35 (0.84−2.16)
	*p* < .001	*p* < .001	*p* < .001	*p* = .22

Notes.

All bolded aORs are significant at *p* < .05.

Control variables in each model:

Model 1: Sociodemographic variables.

Model 2: Model 1 + exposure to tobacco users (family tobacco use, secondhand smoke, friends’ tobacco use).

Model 3: Model 2 + cigarette susceptibility.

Model 4: Model 3 + behavioral risk factors (ever use of other tobacco products, past 12-month use of alcohol and marijuana).

Table 4 reports comparable results for past 30-day cigarette smoking. The association between ever e-cigarette use and smoking declines steadily and substantially from Model 1 to Model 4. Except for waves 3–4, in which the aOR is significant at 2.16 (95% CI, 1.18−3.97), the Model 4 aORs are all non-significant. Specifically, the non-significant aORs in Model 4 for the other wave comparisons are: 1.41 (95% CI, 0.64−3.09) in waves 1–2, 1.41 (95% CI, 0.67−2.98) in waves 2–3, 1.11 (95% CI, 0.57−2.16) in waves 4–4.5, and 1.21 (95% CI, 0.59−2.48) in waves 4.5–5. Full regression results with past 12-month and past 30-day cigarette smoking as outcomes are in Supplement eTables 4A–E and 5A–E.

**Table 4.** Weighted Association of Ever E-Cig Use (Initial Wave) With Subsequent Past 30-Day Cigarette Use (Next Wave) Among US Youth in the PATH Study

	Reported subsequent past 30-day cigarette smoking			
Waves	Model 1 aOR (95% CI)	Model 2 aOR (95% CI)	Model 3 aOR (95% CI)	Model 4 aOR (95% CI)
Waves 1–2	**4.32** **(2.42**−**7.70)**	**3.74** **(2.04**−**6.84)**	**2.45** **(1.36**−**4.42)**	1.41 (0.64−3.09)
	*p* < .001	*p* < .001	*p* = .003	*p* = .39
Waves 2–3	**5.71** **(3.11**−**10.49)**	**3.36** **(1.84**−**6.13)**	**2.80** **(1.58**−**4.96)**	1.41 (0.67−2.98)
	*p* < .001	*p* < .001	*p* = .001	*p* = .37
Waves 3–4	**6.04** **(3.89**−**9.36)**	**3.72** **(2.43**−**5.68)**	**3.04** **(2.00**−**4.63)**	**2.16** **(1.18**−**3.97)**
	*p* < .001	*p* < .001	*p* < .001	*p* = .01
Waves 4–4.5	**4.14** **(2.71**−**6.32)**	**2.71** **(1.72**−**4.26)**	**2.30** **(1.45**−**3.66)**	1.11 (0.57−2.16)
	*p* < .001	*p* < .001	*p* = .001	*p* = .75
Waves 4.5–5	**3.26** **(1.81**−**5.86)**	**1.78** **(1.01**−**3.11)**	1.53 (0.87−2.69)	1.21 (0.59−2.48)
	*p* < .001	*p* = .05	*p* = .14	*p* = .60

Notes.

All bolded aORs are significant at *p* < .05.

Control variables in each model:

Model 1: Sociodemographic variables.

Model 2: Model 1 + exposure to tobacco users (family tobacco use, secondhand smoke, friends’ tobacco use).

Model 3: Model 2 + cigarette susceptibility.

Model 4: Model 3 + behavioral risk factors (ever use of other tobacco products, past 12-month use of alcohol and marijuana).

## Discussion

We found that the significant association observed in previous studies^6–22^ between e-cigarette use and subsequent 12-month smoking remained in the first three consecutive waves (waves 1–2, 2–3, and 3–4) in all four models in our analysis. However, results from the most recent waves (waves 4–4.5 and 4.5–5) indicate that this significant association disappears in Model 4. Adjusting for a full set of confounders, including adolescents’ sociodemographic characteristics, exposure to tobacco users, susceptibility to smoking, and behavioral risk factors, we found that the association of ever e-cigarette use with subsequent smoking decreases substantially and even becomes non-significant in some waves, using both past 12-month and past 30-day smoking as outcomes. We believe this is the first study to report any non-significant findings, likely the result of the more comprehensive set of risk factor variables we included. As we demonstrate with the use of four models, each adding significant risk factor variables (and in turn decreasing the aOR of e-cigarette use), inclusion of logical risk factors is important to interpreting the association between adolescent vaping and subsequent smoking.

The omission of important potential risk factors (such as marijuana use and other tobacco product use) might explain the significant associations between e-cigarette use and subsequent cigarette smoking reported in other studies. Note that for past 12-month smoking (the most common measure of smoking in previous studies), our reported aORs in Model 1 range from 4.07–5.93 and in Model 2 from 2.28 to 4.98. These findings, with susceptibility to smoking and other substance use (and exposure to tobacco users, in the case of Model 1) omitted, are similar to the pooled results reported by Khouja et al.^24^ and Soneji et al.,^23^ 4.59 (95% CI, 3.60−5.85) and 3.50 (95% CI, 2.38−5.16), respectively. One previous study^21^ found that adding cigarette susceptibility or ever use of marijuana produced non-significant associations between ever e-cigarette use and subsequent past 30-day cigarette use (their supplemental eTable 4). However, while the authors stated that inclusion of these variables reduced the magnitude of the vaping-to-smoking odds ratio, they did not acknowledge that the relationship became non-significant.

With regard to 12-month smoking, there are a few possible explanations for the change over time in the significance of the vaping-to-smoking association in our most comprehensive model (significant in the first three wave comparisons and non-significant in the last two). First, vaping prevalence greatly increased during our study period (2013–2019). Prevalence of current vaping among high school students increased from 4.5%^39^ in 2013 to 27.5%^3^ in 2019. There are also changes in vapers and vaping products over time. Given the substantially increased popularity of e-cigarettes, early and later adopters of vaping might have different characteristics such as socioeconomic status, personalities and risk-seeking behaviors, all of which could influence subsequent trial of cigarettes. Vaping devices have also gone through rapid evolution, from the first generation devices of cig-a-likes to the third and fourth generation mod and pod devices.^40^ It is conceivable that these changes in e-cigarette devices have led to changes over time in the demographic and behavioral characteristics of adolescent vapers, modifying the vaping-to-smoking association. Alternatively (or as well), it is possible that as e-cigarettes have become better nicotine delivery devices in recent years, young people were less inclined to try other nicotine products, including cigarettes. Other studies of youth e-cigarette users may have additional plausible explanations.

Using prospective data and the same approach of multivariable logistic regressions as in previous studies, we have examined the longitudinal association between vaping and smoking. Although we do not directly investigate a possible causal relationship between vaping and smoking, our analysis, adjusting for an extensive list of confounders, can provide evidence to support whether such a relationship exists.^41^ As Khouja et al. pointed out, none of the prospective studies they examined satisfied all four preselected Bradford-Hill criteria for causality.^24^ Our study does not meet all four criteria either, but we are the first study to provide some statistical evidence consistent with the common liability theory.

This said, this new evidence cannot be interpreted as “proving” the common liability theory. In the most recent waves (waves 4–4.5 and 4.5–5), we report no direct association between ever vaping and subsequent cigarette smoking among adolescents in our Model 4. It is possible, however, that vaping behaviors could affect cigarette smoking through other channels indirectly.^42^ There are complex causal pathways operating here, and the direction of these may vary over time. Randomized controlled trials are generally considered the strongest study design in examining causal inferences, but they are unethical in this case. There are a few challenges in revealing these pathways using observational data. First, as with many other longitudinal datasets, PATH does not permit examination of the order of risky behaviors, making it impossible to identify any proposed sequence of causality. Even if it did, this would not resolve the issue of causality. Unmeasured confounders, such as a particular personality type or an underlying mental health issue, may affect both vaping and smoking, causing youth to start experimenting with the product that is more readily available, convenient, or socially acceptable. This said, the question is sufficiently important that future research should strive to characterize plausible causal pathways and attempt to assess which one(s) receive strong empirical support.

With regard to the prospective study literature, precisely because some of our results differ from those of the previous studies,^6–22^ future research is needed to either support or refute our findings. However, the critical question about the vaping-to-smoking association is not whether e-cigarette use leads to trial of cigarettes—the issue on which virtually all studies have focused to date, including this one—but rather whether the popularity of vaping will eventually increase smoking. In their recent study, Pierce and colleagues^43^ concluded that, “The recent large increase in e-cigarette use will likely reverse the decline in cigarette smoking among US young adults.” To date, the evidence does not support that conclusion: youth and young adult smoking have declined rapidly during the era of youth vaping.^44,45^ Further, Shahab and colleagues^46^ estimated that less than 1% of US adolescents who initiated nicotine or tobacco use with e-cigarettes became established smokers. None of these studies is the proverbial crystal ball. We need to closely monitor adolescent vaping and smoking behaviors going forward.

### Limitations

We were unable to include one risk factor found in many other studies: a psychosocial measure of sensation seeking. PATH includes questions on sensation seeking but poses them only to new participants in each wave. As such, we could not get a consistent measure for respondents across waves. Given our inclusion of both a measure of susceptibility to smoking and use of other psychoactive substances, this omission would be unlikely to affect our findings.

We excluded from our analysis individuals who aged out of the follow-up adolescent survey by having turned 18. The vaping-to-smoking association might change in magnitude or statistical significance with these aged-up young adults added.

The longitudinal nature of PATH data generates a potential selection bias because certain participants contributed to multiple waves of data. This bias should be attenuated in our study, however, since (1) with sample weights, we created nationally representative samples; (2) we do not compare precise empirical findings across sets of waves; and (3) a replenishment sample was added in wave 4, constituting 40% of that year’s sample.

Another limitation is the potential issue of overfitting with our added control variables. In Supplement eTable 6, we list the limiting sample size (number of occurrences of the less common event) for each wave of our analysis, using past 12-month and past 30-day cigarette smoking as the outcome, respectively. The limiting sample sizes for past 12-month cigarette use exceed the minimal numbers needed for logistic regressions to produce good estimates. However, non-significant results from past 30-day cigarette use may reflect overfitting due to sample size limitations.^47^

Also, our findings depend on the reliability and validity of PATH’s self-reported data. Tourangeau et al.^48^ re-interviewed a subset of adults and youth from PATH wave 4 and then followed self-report responses with saliva samples, concluding that the self-report data on tobacco use are both reliable and valid. More generally, Bachman et al.^49^ (pp. 42–45) reviewed a substantial body of evidence regarding multiple surveys of youth self-reporting of drug use, including tobacco use, and found that the evidence supports the validity of the self-reported data.

## Conclusions

We found that among adolescent never-smokers, those who had ever used e-cigarettes at baseline, compared with never e-cigarette users, exhibited modest or non-significant increases in subsequent smoking when we adjusted for a more complete set of risk factors. These findings offer a new empirical perspective on the debate as to whether e-cigarette use by adolescents is associated with subsequent cigarette use.

## Supplementary Material

ntab243_suppl_Supplementary_Materials

ntab243_suppl_Supplementary_Taxonomy_Form

## Data Availability

Public use files can be accessed at https://www.icpsr.umich.edu/web/NAHDAP/studies/36498.

## References

[1] Gentzke AS , Wang TW, Jamal A, et al. Tobacco product use among middle and high school students—United States, 2020. MMWR Morb Mortal Wkly Rep. 2020;69(50):1881–1888. 33332300 10.15585/mmwr.mm6950a1 PMC7745956

[2] Wang TW , Gentzke AS, Creamer MR, et al. Tobacco product use and associated factors among middle and high school students—United States, 2019. MMWR Surveil Summ. 2019;68(12):1–22. 10.15585/mmwr.ss6812a1 PMC6903396 31805035

[3] Park-Lee E , Ren C, Sawdey MD, et al. Notes from the field: e-cigarette use among middle and high school students—National Youth Tobacco Survey, United States, 2021. MMWR Morb Mortal Wkly Rep. 2021;70(39):1387–1389. 34591834 10.15585/mmwr.mm7039a4 PMC8486384

[4] Eastwood B , Dockrell MJ, Arnott D, et al. Electronic cigarette use in young people in Great Britain 2013–2014. Public Health. 2015;129(9):1150–1156. 26293814 10.1016/j.puhe.2015.07.009

[5] Aleyan S , Cole A, Qian W, Leatherdale ST. Risky business: a longitudinal study examining cigarette smoking initiation among susceptible and non-susceptible e-cigarette users in Canada. BMJ Open. 2018;8(5):e021080. 10.1136/bmjopen-2017-021080 PMC5988055 29804064

[6] Barrington-Trimis JL , Urman R, Berhane K, et al. E-cigarettes and future cigarette use. Pediatrics. 2016;138(1):e20160379. 27296866 10.1542/peds.2016-0379 PMC4925085

[7] Barrington-Trimis JL , Kong G, Leventhal AM, et al. E-cigarette use and subsequent smoking frequency among adolescents. Pediatrics. 2018;142(6):e20180486. 30397165 10.1542/peds.2018-0486 PMC6312103

[8] Best C , Haseen F, Currie D, et al. Relationship between trying an electronic cigarette and subsequent cigarette experimentation in Scottish adolescents: a cohort study. Tob Control. 2018;27(4):373–378. 10.1136/tobaccocontrol-2017-053691 PMC6047138 28735273

[9] Conner M , Grogan S, Simms-Ellis R, et al. Do electronic cigarettes increase cigarette smoking in UK adolescents? Evidence from a 12-month prospective study. Tob Control. 2018;27(4):365–72. 10.1136/tobaccocontrol-2016-053539 PMC6047139 28818839

[10] East K , Hitchman SC, Bakolis I, et al. The association between smoking and electronic cigarette use in a cohort of young people. J Adolesc Health. 2018;62(5):539–547. 29499983 10.1016/j.jadohealth.2017.11.301 PMC5938086

[11] Hammond D , Reid JL, Cole AG, Leatherdale ST. Electronic cigarette use and smoking initiation among youth: a longitudinal cohort study. CMAJ. 2017;189(43):E1328–E1336. 29084759 10.1503/cmaj.161002 PMC5662449

[12] Leventhal AM , Strong DR, Kirkpatrick MG, et al. Association of electronic cigarette use with initiation of combustible tobacco product smoking in early adolescence. JAMA. 2015;314(7):700–707. 26284721 10.1001/jama.2015.8950 PMC4771179

[13] Loukas A , Marti CN, Cooper M, Pasch KE, Perry CL. Exclusive e-cigarette use predicts cigarette initiation among college students. Addict Behav. 2018;76:343–347. 28892771 10.1016/j.addbeh.2017.08.023 PMC5614895

[14] Lozano P , Barrientos-Gutierrez I, Arillo-Santillan E, et al. A longitudinal study of electronic cigarette use and onset of conventional cigarette smoking and marijuana use among Mexican adolescents. Drug Alcohol Depend. 2017;180:427–430. 28988005 10.1016/j.drugalcdep.2017.09.001 PMC5771440

[15] Miech R , Patrick ME, O’Malley PM, Johnston LD. E-cigarette use as a predictor of cigarette smoking: results from a 1-year follow-up of a national sample of 12^th^ grade students. Tob Control. 2017;26(e2):e106–e111. 28167683 10.1136/tobaccocontrol-2016-053291 PMC5545162

[16] Morgenstern M , Nies A, Goecke M, Hanewinkel R. E-Cigarettes and the use of conventional cigarettes. Dtsch Arztebl Int. 2018;115(14):243–248. 29716689 10.3238/arztebl.2018.0243 PMC5938547

[17] Primack BA , Soneji S, Stoolmiller M, Fine MJ, Sargent JD. Progression to traditional cigarette smoking after electronic cigarette use among US adolescents and young adults. JAMA Pediatr. 2015;169(11):1018–1023. 26348249 10.1001/jamapediatrics.2015.1742 PMC4800740

[18] Primack BA , Shensa A, Sidani JE, et al. Initiation of traditional cigarette smoking after electronic cigarette use among tobacco-naïve US young adults. Am J Med. 2018;131(4):443.e1–443.e9. 10.1016/j.amjmed.2017.11.005 PMC7054856 29242110

[19] Spindle TR , Hiler MM, Cooke ME, Eissenberg T, Kendler KS, Dick DM. Electronic cigarette use and uptake of cigarette smoking: a longitudinal examination of U.S. college students. Addict Behav. 2017;67:66–72. 28038364 10.1016/j.addbeh.2016.12.009 PMC5250543

[20] Treur JL , Rozema AD, Mathijssen JJP, van Oers H, Vink JM. E-cigarette and waterpipe use in two adolescent cohorts: cross-sectional and longitudinal associations with conventional cigarette smoking. Eur J Epidemiol. 2018;33(3):323–334. 29260431 10.1007/s10654-017-0345-9 PMC5889768

[21] Watkins SL , Glantz SA, Chaffee BW. Association of noncigarette tobacco product use with future cigarette smoking among youth in the Population Assessment of Tobacco and Health (PATH) study, 2013–2015. JAMA Pediatr. 2018;172(2):181–187. 29297010 10.1001/jamapediatrics.2017.4173 PMC5801043

[22] Wills TA , Knight R, Williams RJ, Pagano I, Sargent JD. Risk factors for exclusive e-cigarette use and dual e-cigarette use and tobacco use in adolescents. Pediatrics. 2015;135(1):e43–e51. 25511118 10.1542/peds.2014-0760 PMC4279062

[23] Soneji S , Barrington-Trimis JL, Wills TA, et al. Association between initial use of e-cigarettes and subsequent cigarette smoking among adolescents and young adults: a systematic review and meta-analysis. JAMA Pediatr. 2017;171(8):788–797. 28654986 10.1001/jamapediatrics.2017.1488 PMC5656237

[24] Khouja JN , Suddell SF, Peters SE, Taylor AE, Munafò MR. Is e-cigarette use in non-smoking young adults associated with later smoking? A systematic review and meta-analysis. Tob Control. 2021;30(1):8–15. 10.1136/tobaccocontrol-2019-055433 PMC7803902 32156694

[25] Bell K , Keane H. All gates lead to smoking: the ‘gateway theory’, e-cigarettes and the remaking of nicotine. Soc Sci Med. 2014;119:45–52. 25150650 10.1016/j.socscimed.2014.08.016

[26] National Academies of Sciences, Engineering, and Medicine 2018. Public Health Consequences of E-Cigarettes. Washington, DC: The National Academies Press. 29894118

[27] Glasser A , Abudayyeh H, Cantrell J, Niaura R. Patterns of e-cigarette use among youth and young adults: review of the impact of e-cigarettes on cigarette smoking. Nicotine Tob Res. 2019;21(10):1320–1330. 29788314 10.1093/ntr/nty103

[28] Etter JF . Gateway effects and electronic cigarettes. Addiction. 2018;113(10):1776–1783. 28786147 10.1111/add.13924

[29] Chapman S , Bareham D, Maziak W. The gateway effect of e-cigarettes: reflections on main criticisms. Nicotine Tob Res. 2019;21(5):695–698. 29660054 10.1093/ntr/nty067 PMC6468127

[30] Kim S , Selya AS. The relationship between electronic cigarette use and conventional cigarette smoking is largely attributable to shared risk factors. Nicotine Tob Res. 2020;22(7):1123–1130. 31680169 10.1093/ntr/ntz157 PMC7291806

[31] Glantz SA . Convincing analysis that e-cigs are a gateway to cigarette smoking from studies around the world. UCSF Center for Tobacco Control Research and Education. https://tobacco.ucsf.edu/convincing-analysis-e-cigs-are-gateway-cigarette-smoking-studies-around-world (Accessed October 23, 2021).

[32] Kozlowski LT , Abrams DB. Obsolete tobacco control themes can be hazardous to public health: the need for updating views on absolute product risks and harm reduction. BMC Public Health. 2016;16:432. 27221096 10.1186/s12889-016-3079-9 PMC4878038

[33] Kozlowski LT , Warner KE. Adolescents and e-cigarettes: objects of concern may appear larger than they are. Drug Alcohol Depend. 2017;174:209–214. 29350617 10.1016/j.drugalcdep.2017.01.001

[34] Lee P , Fry J. Investigating gateway effects using the PATH study. F1000Res. 2019;8:264. 31956397 10.12688/f1000research.18354.2 PMC6950312

[35] Hyland A , Ambrose BK, Conway KP, et al. Design and methods of the Population Assessment of Tobacco and Health (PATH) study. Tob Control. 2017;26(4):371–378. 27507901 10.1136/tobaccocontrol-2016-052934 PMC5299069

[36] WeStat. PATH Study public use files: user guide. 2020. https://www.icpsr.umich.edu/web/NAHDAP/studies/37786 (Accessed October 23, 2021).

[37] Pierce JP , Choi WS, Gilpin EA, Farkas AJ, Merritt RK. Validation of susceptibility as a predictor of which adolescents take up smoking in the United States. Health Psychol. 1996;15(5):355–361. 8891714 10.1037//0278-6133.15.5.355

[38] Nicksic NE , Barnes AJ. Is susceptibility to E-cigarettes among youth associated with tobacco and other substance use behaviors one year later? Results from the PATH study. Prev Med. 2019;121:109–114. 30776386 10.1016/j.ypmed.2019.02.006 PMC6594855

[39] Arrazola RA , Neff LJ, Kennedy SM, Holder-Hayes E, Jones CD; Centers for Disease Control and Prevention (CDC). Tobacco use among middle and high school students–United States, 2013. MMWR Morb Mortal Wkly Rep. 2014;63(45):1021–1026. 25393220 PMC5779497

[40] Williams M , Talbot P. Design features in multiple generations of electronic cigarette atomizers. Int J Environ Res Public Health. 2019;16(16):2904. 10.3390/ijerph16162904 PMC6720609 31416115

[41] Hernán MA . The C-Word: scientific Euphemisms do not improve causal inference from observational data. Am J Public Health. 2018;108(5):616–619. 29565659 10.2105/AJPH.2018.304337 PMC5888052

[42] Wills TA , Gibbons FX, Sargent JD, Schweitzer RJ. How is the effect of adolescent e-cigarette use on smoking onset mediated: a longitudinal analysis. Psychol Addict Behav. 2016;30(8):876–886. 27669093 10.1037/adb0000213 PMC5222747

[43] Pierce JP , Chen R, Leas EC, et al. Use of E-cigarettes and other tobacco products and progression to daily cigarette smoking. Pediatrics. 2021;147(2):e2020025122. 33431589 10.1542/peds.2020-025122 PMC7849197

[44] Levy DT , Warner KE, Cummings KM, et al. Examining the relationship of vaping to smoking initiation among US youth and young adults: a reality check. Tob Control. 2019;28(6):629–635. 30459182 10.1136/tobaccocontrol-2018-054446 PMC6860409

[45] Meza R , Jimenez-Mendoza E, Levy DT. Trends in Tobacco use among Adolescents by Grade, Sex, and Race, 1991–2019. JAMA Netw Open. 2020;3(12):e2027465. 33263760 10.1001/jamanetworkopen.2020.27465 PMC7711321

[46] Shahab L , Beard E, Brown J. Association of initial e-cigarette and other tobacco product use with subsequent cigarette smoking in adolescents: a cross-sectional, matched control study. Tob Control. 2021;30(2):212–220. 32184339 10.1136/tobaccocontrol-2019-055283 PMC7907552

[47] Peduzzi P , Concato J, Feinstein AR, Holford TR. Importance of events per independent variable in proportional hazards regression analysis. II. Accuracy and precision of regression estimates. J Clin Epidemiol. 1995;48(12):1503–1510. 8543964 10.1016/0895-4356(95)00048-8

[48] Tourangeau R , Yan T, Sun H, Hyland A, Stanton CA. Population Assessment of Tobacco and Health (PATH) reliability and validity study: selected reliability and validity estimates. Tob Control. 2019;28(6):663–668. 30297373 10.1136/tobaccocontrol-2018-054561

[49] Bachman JG , Johnston LD, O’Malley PM, Schulenberg JE, Miech RA. The Monitoring The Future Project after four decades: design and procedures. Monitoring the Future Occasional Paper Series, Paper #82. Ann Arbor, MI: University of Michigan, Institute for Social Research, 2015. http://www.monitoringthefuture.org/pubs/occpapers/mtf-occ82.pdf (Accessed October 23, 2021).

