# Supplementary material for: Is Adolescent E-Cigarette Use Associated With Subsequent Smoking? A New Look
Source: Nicotine Tob Res. 2021 Nov 20;24(5):710–8. doi: 10.1093/ntr/ntab243 (PMC8962683; doi:10.1093/ntr/ntab243)
Supplement: ntab243_suppl_Supplementary_Materials [file ntab243_suppl_supplementary_materials.docx]

**Supplement to Sun et al.**

**Is Adolescent E-Cigarette Use Associated With Subsequent Smoking? A New Look**

eTable 1. Association between ever e-cigarette use at baseline and subsequent smoking initiation among never cigarette smokers in previous studies.

eTables 2A-D. Sample characteristics among never cigarette smokers at baseline (PATH Study waves 1-4).

eTables 3A-D. Weighted proportions of baseline never cigarette smokers using cigarettes in subsequent wave by risk factors (excluding sociodemographic characteristics).

eTables 4A-E. Weighted association of ever e-cig use (initial wave) with subsequent past 12-month cigarette use (latter wave) among US youth in the PATH Study - 4 Models with full regression results.

eTables 5A-E. Weighted association of ever e-cig use (initial wave) with subsequent past 30-day cigarette use (latter wave) among US youth in the PATH Study - 4 Models with full regression results.

eTable 6. Limiting sample size of outcome variables (past 12-month and past 30-day cigarette use) without survey weights.

eTable 1. Association between ever e-cigarette use at baseline and subsequent smoking initiation among never cigarette smokers in previous studies.*

| Authors | Year | aOR**  (95% CI) | Age  (years) | Sample Size | Demo-  graphics^a^ | Psycho-social^b^ | Susceptibility^c^ | Exposure to Tobacco Users^d^ | Other tobacco use^e^ | Other drug use^f^ | Other^g^ |
| --- | --- | --- | --- | --- | --- | --- | --- | --- | --- | --- | --- |
| Barrington-Trimis et al.^6^ | 2016 | 6.17 (3.29-11.57) | 16-18 | 146 | X |  | X | X |  |  | X |
| Barrington-Trimis et al.^7^ | 2018 | 4.58 (3.56-5.88) | 16-18 | 6,258 | X |  |  |  |  |  | X |
| Best et al.^8^ | 2018 | 2.42 (1.63-3.60) | 11-18 | 2,125 | X |  | X | X |  |  | X |
| Conner et al.^9^ | 2018 | 4.06 (2.94-5.60) | 13-14 | 1,726 | X | X |  | X |  |  | X |
| East et al.^10^ | 2018 | 10.57 (3.33-33.50) | 11-18 | 923 | X |  | X | X |  | X | X |
| Hammond et al.^11^*** | 2017 | 2.12 (1.68-2.66) | 14-18 | 17,318 | X |  | X |  |  |  | X |
| Leventhal et al.^12^ | 2015 | 1.75 (1.10-2.78) | 14-15 | 2,530 | X | X | X | X |  | X | X |
| Loukas et al.^13^ | 2018 | 1.36 (1.01-1.83) | 18-25 | 2,558 | X |  | X | X | X |  |  |
| Lozano et al.^14^ | 2017 | 1.40 (1.22-1.60) | 12-13 | 6,574 | X | X |  | X |  | X | X |
| Miech et al.^15^ | 2017 | 4.78 (1.91-11.96) | 17-18 | 347 | X |  |  |  |  | X |  |
| Morgenstern et al.^16^ | 2018 | 2.50 (1.82-3.54) | 15-16 | 2,186 | X | X |  |  |  | X | X |
| Primack et al.^17^ | 2015 | 8.30 (1.20-58.60) | 16-26 | 694 | X | X |  | X |  |  | X |
| Primack et al.^18^ | 2018 | 6.82 (1.65-28.25) | 18-30 | 915 | X | X |  |  |  |  | X |
| Spindle et al.^19^ | 2017 | 3.37 (1.91-5.94) | 18-19 | 3,757 | X | X |  |  | X |  |  |
| Treur et al.^20^ | 2018 | 11.90 (3.36-42.11) | 14-21 | 2,100 | X | X |  |  |  |  | X |
| Watkins et al.^21^ | 2018 | 2.53 (1.80-3.56) | 12-17 | 10,384 | X | X |  | X | X | X | X |
| Wills et al.^22^ | 2017 | 2.87 (2.03-4.05) | 14-16 | 2,338 | X | X |  |  |  |  | X |

Note

* We included 17 studies that used longitudinal data from meta-analyses conducted by Khouja et al.^23^ and Soneji et al.^22^ All studies except one (Ref 5) were in Khouja et al.^23^ References 11, 14, 16, 18, and 21 were included in both meta-analyses.

** aORs were summarized or derived by Khouja et al.^23^ and Soneji et al.^22^

***Examined the association between past 30-day vaping at baseline with subsequent smoking initiation.

a Demographic variables include age, sex, and race/ethnicity.

b Examples of psychosocial measures include intentions, attitude, perceived norms, sensation seeking, personality traits, self-efficacy, self-esteem, rebelliousness, and impulsivity.

c Susceptibility includes smoking/cigarette susceptibility measure.

d Exposure to tobacco users is assessed by peer influence, friends smoking, family smoking, family tobacco use, and secondhand smoke.

e Other tobacco use is measured by ever use of other tobacco products, such as smokeless tobacco/snus, little cigars/cigarillos, and hookah.

f Other drug use controls for substance use such as alcohol, marijuana, and other illicit drugs.

g Other variables include parental education, school grade, household income, urban residency, and living arrangement.

eTable 2A. Sample characteristics among never cigarette smokers at baseline, PATH Study wave 1 (N = 9,045).

|  | **Never Cigarette Smokers by Wave 1** | |
| --- | --- | --- |
|  | **Proportion (%)** | **Weighted Proportion (%, 95% CI)** |
| **Sociodemographic Characteristics** | |  |
| *Sex* |  |  |
| Male | 51.3 | 51.3 (50.8-51.8) |
| Female | 48.7 | 48.7 (48.3-49.2) |
| *Age* |  |  |
| 12-14 | 64.5 | 64.1 (63.6-64.6) |
| 15-17 | 35.5 | 35.9 (35.4-36.4) |
| *Race/Ethnicity* |  |  |
| Non-Hispanic white | 47.5 | 54.0 (53.3-54.6) |
| Non-Hispanic black | 13.9 | 14.1 (13.7-14.5) |
| Hispanic | 29.5 | 22.7 (22.3-23.2) |
| Non-Hispanic other | 9.1 | 9.3 (8.9-9.7) |
| *Highest parental education* |  |  |
| High school/GED or less | 37.8 | 33.9 (32.1-35.7) |
| Some college | 31.7 | 31.5 (29.8-33.2) |
| College or higher | 30.5 | 34.6 (32.3-37.0) |
| *Grades > mostly B’s* |  |  |
| Yes | 71.5 | 73.2 (72.1-74.2) |
| No | 28.5 | 26.8 (25.8-27.9) |
| **Exposure to Tobacco Users** |  |  |
| *Family tobacco use* |  |  |
| Yes | 31.7 | 30.7 (28.9-32.4) |
| No | 68.3 | 69.4 (67.6-71.1) |
| *Secondhand smoke* |  |  |
| Yes | 35.1 | 34.6 (33.4-35.9) |
| No | 65.0 | 65.4 (64.2-66.6) |
| **Susceptibility** |  |  |
| *Susceptible to cigarettes* |  |  |
| Yes | 32.3 | 32.2 (31.1-33.2) |
| No | 67.7 | 67.8 (66.8-68.9) |
| **Behavioral Risk Factors** |  |  |
| *Ever vaped* |  |  |
| Yes | 3.7 | 3.7 (3.3-4.1) |
| No | 96.3 | 96.3 (95.9-96.7) |
| *Ever used other tobacco products** | | |
| Yes | 4.8 | 4.7 (4.2-5.3) |
| No | 95.3 | 95.3 (94.7-95.8) |
| *Used alcohol in past 12 months* |  |  |
| Yes | 10.4 | 10.6 (9.8-11.5) |
| No | 89.6 | 89.4 (88.5-90.2) |
| *Used marijuana in past 12 months* |  |  |
| Yes | 1.8 | 1.7 (1.4-2.0) |
| No | 98.3 | 98.3 (9.0-98.6) |

* Other tobacco products include cigar, pipe, hookah, snus, smokeless tobacco, bidi, kretek, and dissolvable tobacco.

eTable 2B. Sample characteristics among never cigarette smokers at baseline, PATH Study wave 2 (N = 8,668).

|  | **Never Cigarette Smokers by Wave 2** | |
| --- | --- | --- |
|  | **Proportion (%)** | **Weighted Proportion (%, 95% CI)** |
| **Sociodemographic Characteristics** | |  |
| *Sex* |  |  |
| Male | 51.6 | 51.4 (50.8-51.9) |
| Female | 48.4 | 48.6 (48.1-49.2) |
| *Age* |  |  |
| 12-14 | 63.3 | 62.7 (62.2-63.2) |
| 15-17 | 36.7 | 37.3 (36.8-37.8) |
| *Race/Ethnicity* |  |  |
| Non-Hispanic white | 46.8 | 52.6 (52.0-53.2) |
| Non-Hispanic black | 13.7 | 13.9 (13.5-14.3) |
| Hispanic | 30.3 | 23.7 (23.3-24.2) |
| Non-Hispanic other | 9.3 | 9.8 (9.5-10.2) |
| *Highest parental education* |  |  |
| High school/GED or less | 37.4 | 33.3 (31.6-35.2) |
| Some college | 31.1 | 30.5 (28.9-32.1) |
| College or higher | 31.5 | 36.2 (33.9-38.6) |
| *Household income* |  |  |
| < 50k | 50.6 | 46.0 (44.1-47.8) |
| 50k to 100k | 25.7 | 26.5 (25.2-27.8) |
| > 100k | 23.8 | 27.6 (25.5-29.8) |
| *Grades > mostly B’s* |  |  |
| Yes | 71.2 | 73.0 (71.8-74.1) |
| No | 28.8 | 27.0 (25.9-28.2) |
| **Exposure to Tobacco Users** |  |  |
| *Family tobacco use* |  |  |
| Yes | 29.0 | 28.0 (26.5-29.6) |
| No | 71.0 | 72.0 (70.5-73.5) |
| *Secondhand smoke* |  |  |
| Yes | 31.1 | 31.0 (29.5-32.5) |
| No | 68.9 | 69.0 (67.5-70.5) |
| *Friends’ tobacco use* |  |  |
| Yes | 27.5 | 26.8 (25.7-27.9) |
| No | 72.5 | 73.2 (72.1-74.3) |
| **Susceptibility** |  |  |
| *Susceptible to cigarettes* |  |  |
| Yes | 29.3 | 29.0 (28.0-30.1) |
| No | 70.7 | 71.0 (69.9-72.0) |
| **Behavioral Risk Factors** |  |  |
| *Ever vaped* |  |  |
| Yes | 9.0 | 8.7 (8.2-9.3) |
| No | 91.0 | 91.3 (90.7-91.9) |
| *Ever used other tobacco products* | | |
| Yes | 4.9 | 4.9 (4.4-5.4) |
| No | 95.1 | 95.1 (94.6-95.6) |
| *Used alcohol in past 12 months* |  |  |
| Yes | 16.6 | 17.2 (16.2-18.3) |
| No | 83.4 | 82.8 (81.7-83.8) |
| *Used marijuana in past 12 months* |  |  |
| Yes | 2.2 | 2.1 (1.8-2.5) |
| No | 97.8 | 97.9 (97.5-98.2) |

eTable 2C. Sample characteristics among never cigarette smokers at baseline, PATH Study wave 3 (N = 8,294).

|  | **Never Cigarette Smokers by Wave 3** | |
| --- | --- | --- |
|  | **Proportion (%)** | **Weighted Proportion (%, 95% CI)** |
| **Sociodemographic Characteristics** | |  |
| *Sex* |  |  |
| Male | 51.4 | 50.9 (50.3-51.5) |
| Female | 48.6 | 48.8 (48.2-49.4) |
| *Age* |  |  |
| 12-14 | 63.8 | 63.8 (63.1-64.5) |
| 15-17 | 36.2 | 36.2 (35.6-36.9) |
| *Race/Ethnicity* |  |  |
| Non-Hispanic white | 44.1 | 49.6 (48.9-50.4) |
| Non-Hispanic black | 13.4 | 13.3 (12.8-13.8) |
| Hispanic | 29.5 | 23.5 (22.9-24.1) |
| Non-Hispanic other | 9.0 | 9.7 (9.2-10.2) |
| *Highest parental education* |  |  |
| High school/GED or less | 36.4 | 32.2 (30.5-33.9) |
| Some college | 31.5 | 31.0 (29.3-32.6) |
| College or higher | 32.1 | 36.9 (34.7-39.1) |
| *Household income* |  |  |
| < 50k | 48.6 | 43.6 (41.7-45.5) |
| 50k to 100k | 25.9 | 26.9 (25.5-28.5) |
| > 100k | 25.5 | 29.5 (27.4-31.7) |
| *Grades > mostly B’s* |  |  |
| Yes | 72.2 | 74.0 (72.8-75.3) |
| No | 27.8 | 26.0 (24.7-27.2) |
| **Exposure to Tobacco Users** |  |  |
| *Family tobacco use* |  |  |
| Yes | 30.2 | 29.6 (28.1-31.2) |
| No | 69.8 | 70.4 (68.8-71.9) |
| *Secondhand smoke* |  |  |
| Yes | 27.4 | 27.3 (25.8-28.7) |
| No | 72.6 | 72.7 (71.3-74.2) |
| *Friends’ tobacco use* |  |  |
| Yes | 24.8 | 24.6 (23.5-25.7) |
| No | 75.2 | 75.4 (74.3-76.5) |
| **Susceptibility** |  |  |
| *Susceptible to cigarettes* |  |  |
| Yes | 29.4 | 29.1 (28.0-30.2) |
| No | 70.6 | 70.9 (69.8-72.0) |
| **Behavioral Risk Factors** |  |  |
| *Ever vaped* |  |  |
| Yes | 8.1 | 7.9 (7.2-8.6) |
| No | 91.9 | 92.1 (91.4-92.8) |
| *Ever used other tobacco products** | | |
| Yes | 4.4 | 4.4 (3.9-4.9) |
| No | 95.6 | 95.6 (95.1-96.1) |
| *Used alcohol in past 12 months* |  |  |
| Yes | 15.8 | 16.6 (15.4-17.8) |
| No | 84.2 | 83.4 (82.2-85.6) |
| *Used marijuana in past 12 months* |  |  |
| Yes | 2.2 | 2.2 (1.9-2.6) |
| No | 97.9 | 97.8 (97.4-98.1) |

eTable 2D. Sample characteristics among never cigarette smokers at baseline, PATH Study wave 4 (N = 10,208).

|  | **Never Cigarette Smokers by Wave 4** | |
| --- | --- | --- |
|  | **Proportion (%)** | **Weighted Proportion (%, 95% CI)** |
| **Sociodemographic Characteristics** | |  |
| *Sex* |  |  |
| Male | 51.8 | 50.8 (50.4-51.3) |
| Female | 48.2 | 49.2 (48.7-49.6) |
| *Age* |  |  |
| 12-14 | 62.7 | 63.1 (62.7-63.5) |
| 15-17 | 37.3 | 36.9 (36.5-37.3) |
| *Race/Ethnicity* |  |  |
| Non-Hispanic white | 45.9 | 52.2 (51.7-52.7) |
| Non-Hispanic black | 13.8 | 13.6 (13.3-13.9) |
| Hispanic | 30.7 | 24.0 (23.6-24.4) |
| Non-Hispanic other | 9.7 | 10.2 (10.0-10.5) |
| *Highest parental education* |  |  |
| High school/GED or less | 36.0 | 31.9 (30.5-33.2) |
| Some college | 31.0 | 30.5 (29.1-31.9) |
| College or higher | 33.1 | 37.7 (35.9-39.4) |
| *Household income* |  |  |
| < 50k | 46.7 | 41.7 (40.2-43.3) |
| 50k to 100k | 25.5 | 26.2 (25.1-27.4) |
| > 100k | 27.8 | 32.0 (30.4-33.7) |
| *Grades > mostly B’s* |  |  |
| Yes | 72.2 | 73.9 (72.8-74.9) |
| No | 27.8 | 26.1 (25.1-27.2) |
| **Exposure to Tobacco Users** |  |  |
| *Family tobacco use* |  |  |
| Yes | 28.9 | 28.7 (27.4-30.1) |
| No | 71.1 | 71.3 (69.9-72.6) |
| *Secondhand smoke* |  |  |
| Yes | 26.6 | 26.7 (25.5-28.0) |
| No | 73.4 | 73.3 (72.0-74.5) |
| *Friends’ tobacco use* |  |  |
| Yes | 24.1 | 24.0 (22.9-25.1) |
| No | 75.9 | 76.0 (74.9-77.1) |
| **Susceptibility** |  |  |
| *Susceptible to cigarettes* |  |  |
| Yes | 28.4 | 28.2 (27.2-29.3) |
| No | 71.6 | 71.8 (70.7-72.9) |
| **Behavioral Risk Factors** |  |  |
| *Ever vaped* |  |  |
| Yes | 7.4 | 7.3 (6.7-7.9) |
| No | 92.6 | 92.7 (92.1-93.2) |
| *Ever used other tobacco products** | | |
| Yes | 3.7 | 3.7 (3.3-4.1) |
| No | 96.3 | 96.3 (95.9-96.7) |
| *Used alcohol in past 12 months* |  |  |
| Yes | 18.4 | 19.2 (18.2-20.2) |
| No | 81.6 | 80.8 (79.8-81.8) |
| *Used marijuana in past 12 months* |  |  |
| Yes | 2.7 | 2.7 (2.4-3.0) |
| No | 97.3 | 97.4 (97.0-97.7) |

eTable 3A. Weighted proportions of baseline (wave 1) never cigarette smokers using cigarettes in wave 2 by risk factors (excluding sociodemographic characteristics), PATH Study (N = 9,045).

| **Risk Factors** | **Past 12-Month Cigarette Smoking**  **(%, 95% CI)** | **Past 30-Day Cigarette Smoking**  **(%, 95% CI)** |
| --- | --- | --- |
| *Ever vaped** |  |  |
| Yes | 17.8 (13.7-22.9) | 7.1 (4.4-11.2) |
| No | 3.2 (2.8-3.6) | 1.4 (1.1-1.7) |
| **Exposure to Tobacco Users** |  |  |
| *Family tobacco use* |  |  |
| Yes | 5.8 (4.9-6.8) | 2.8 (2.2-3.6) |
| No | 2.8 (2.4-3.3) | 1.0 (0.8-1.3) |
| *Secondhand smoke* |  |  |
| Yes | 6.0 (5.2-7.1) | 2.8 (2.2-3.6) |
| No | 2.6 (2.2-3.1) | 0.9 (0.7-1.2) |
| **Susceptibility** |  |  |
| *Susceptible to cigarettes* | |  |
| Yes | 7.9 (6.8-9.1) | 3.4 (2.7-4.4) |
| No | 1.7 (1.4-2.1) | 0.7 (0.5-0.9) |
| **Behavioral Risk Factors** |  |  |
| *Ever used other tobacco products*** | |  |
| Yes | 13.4 (10.3-17.3) | 5.7 (3.7-8.8) |
| No | 3.3 (2.9-3.7) | 1.4 (1.1-1.7) |
| *Used alcohol in past 12 months* | |  |
| Yes | 13.1 (10.7-15.9) | 4.8 (3.5-6.7) |
| No | 2.6 (2.3-3.0) | 1.2 (1.0-1.5) |
| *Used marijuana in past 12 months* | |  |
| Yes | 19.1 (13.1-27.0) | 8.3 (4.7-14.2) |
| No | 3.5 (3.1-3.8) | 1.4 (1.2-1.7) |

* All risk factors are significant at p<.001 using Pearson’s chi-squared test of independence.

** Other tobacco products include cigar, pipe, hookah, snus, smokeless tobacco, bidi, kretek, and dissolvable tobacco

eTable 3B. Weighted proportions of baseline (wave 2) never cigarette smokers using cigarettes in wave 3 by risk factors (excluding sociodemographic characteristics), PATH Study (N = 8,668).

| **Risk Factors** | **Past 12-Month Cigarette Smoking**  **(%, 95% CI)** | **Past 30-Day Cigarette Smoking**  **(%, 95% CI)** |
| --- | --- | --- |
| *Ever vaped** |  |  |
| Yes | 13.5 (10.7-16.8) | 5.5 (3.8-7.9) |
| No | 2.3 (2.0-2.8) | 0.9 (0.7-1.1) |
| **Exposure to Tobacco Users** |  |  |
| *Family tobacco use* |  |  |
| Yes | 4.9 (4.1-5.9) | 2.1 (1.6-2.9) |
| No | 2.7 (2.3-3.2) | 1.0 (0.7-1.3) |
| *Secondhand smoke* |  |  |
| Yes | 5.8 (4.8-6.9) | 2.6 (1.9-3.5) |
| No | 2.2 (1.8-2.7) | 0.7 (0.5-1.0) |
| *Friends’ tobacco use* |  |  |
| Yes | 7.9 (6.7-9.3) | 3.2 (2.5-4.2) |
| No | 1.6 (1.3-2.0) | 0.5 (0.4-0.8) |
| **Susceptibility** |  |  |
| *Susceptible to cigarettes* | |  |
| Yes | 7.8 (6.8-9.1) | 3.0 (2.3-3.9) |
| No | 1.4 (1.1-1.9) | 0.6 (0.4-0.8) |
| **Behavioral Risk Factors** |  |  |
| *Ever used other tobacco products*** | |  |
| Yes | 13.9 (10.7-17.7) | 7.0 (4.6-10.6) |
| No | 2.8 (2.5-3.2) | 1.0 (0.8-1.2) |
| *Used alcohol in past 12 months* | |  |
| Yes | 8.1 (6.7-9.7) | 3.0 (2.3-4.0) |
| No | 2.3 (1.9-2.8) | 0.9 (0.7-1.2) |
| *Used marijuana in past 12 months* | |  |
| Yes | 15.7 (10.4-22.9) | 7.0 (3.5-13.2) |
| No | 2.7 (2.3-3.1) | 1.0 (0.8-1.3) |

* All risk factors are significant at p<.001 using Pearson’s chi-squared test of independence.

** Other tobacco products include cigar, pipe, hookah, snus, smokeless tobacco, bidi, kretek, and dissolvable tobacco

eTable 3C. Weighted proportions of baseline (wave 3) never cigarette smokers using cigarettes in wave 4 by risk factors (excluding sociodemographic characteristics), PATH Study (N = 8,294).

| **Risk Factors** | **Past 12-Month Cigarette Smoking**  **(%, 95% CI)** | **Past 30-Day Cigarette Smoking**  **(%, 95% CI)** |
| --- | --- | --- |
| *Ever vaped** |  |  |
| Yes | 14.0 (11.6-16.9) | 7.2 (5.5-9.5) |
| No | 2.4 (2.0-2.8) | 1.0 (0.7-1.3) |
| **Exposure to Tobacco Users** |  |  |
| *Family tobacco use* |  |  |
| Yes | 5.5 (4.5-6.6) | 2.6 (2.0-3.4) |
| No | 2.4 (2.0-2.9) | 1.0 (0.7-1.3) |
| *Secondhand smoke* |  |  |
| Yes | 6.3 (5.2-7.6) | 3.0 (2.3-3.8) |
| No | 2.2 (1.8-2.6) | 0.9 (0.7-1.2) |
| *Friends’ tobacco use* |  |  |
| Yes | 7.4 (6.2-8.8) | 3.8 (2.9-4.8) |
| No | 2.0 (1.7-2.3) | 0.7 (0.5-1.0) |
| **Susceptibility** |  |  |
| *Susceptible to cigarettes* | |  |
| Yes | 7.4 (6.3-8.7) | 3.4 (2.7-4.3) |
| No | 1.6 (1.3-2.0) | 0.7 (0.5-1.0) |
| **Behavioral Risk Factors** |  |  |
| *Ever used other tobacco products*** | |  |
| Yes | 14.3 (10.0-19.9) | 5.7 (3.5-9.2) |
| No | 2.8 (2.4-3.2) | 1.3 (1.0-1.6) |
| *Used alcohol in past 12 months* | |  |
| Yes | 8.1 (6.5-10.0) | 3.8 (2.7-5.3) |
| No | 2.3 (2.0-2.8) | 1.1 (0.8-1.3) |
| *Used marijuana in past 12 months* | |  |
| Yes | 16.6 (11.3-23.6) | 10.2 (6.1-16.4) |
| No | 2.6 (2.2-3.0) | 1.1 (0.9-1.4) |

* All risk factors are significant at p<.001 using Pearson’s chi-squared test of independence.

** Other tobacco products include cigar, pipe, hookah, snus, smokeless tobacco, bidi, kretek, and dissolvable tobacco

eTable 3D. Weighted proportions of baseline (wave 4) never cigarette smokers using cigarettes in wave 4.5 by risk factors (excluding sociodemographic characteristics), PATH Study (N = 10,208).

| **Risk Factors** | **Past 12-Month Cigarette Smoking**  **(%, 95% CI)** | **Past 30-Day Cigarette Smoking**  **(%, 95% CI)** |
| --- | --- | --- |
| *Ever vaped** |  |  |
| Yes | 12.0 (9.8-14.5) | 4.9 (3.7-6.5) |
| No | 1.9 (1.6-2.2) | 0.8 (0.6-1.0) |
| **Exposure to Tobacco Users** |  |  |
| *Family tobacco use* |  |  |
| Yes | 4.5 (3.8-5.2) | 2.3 (1.8-2.8) |
| No | 1.9 (1.6-2.3) | 0.6 (0.4-0.8) |
| *Secondhand smoke* |  |  |
| Yes | 4.7 (3.9-5.7) | 2.0 (1.5-2.7) |
| No | 1.9 (1.6-2.3) | 0.7 (0.5-0.9) |
| *Friends’ tobacco use* |  |  |
| Yes | 6.5 (5.6-7.5) | 2.9 (2.3-3.6) |
| No | 1.4 (1.2-1.8) | 0.5 (0.4-0.7) |
| **Susceptibility** |  |  |
| *Susceptible to cigarettes* | |  |
| Yes | 6.0 (5.1-7.0) | 2.3 (1.8-3.0) |
| No | 1.3 (1.1-1.7) | 0.6 (0.4-0.8) |
| **Behavioral Risk Factors** |  |  |
| *Ever used other tobacco products*** | |  |
| Yes | 13.4 (10.3-17.2) | 6.0 (4.0-9.0) |
| No | 2.2 (1.9-2.6) | 0.9 (0.7-1.1) |
| *Used alcohol in past 12 months* | |  |
| Yes | 6.3 (5.3-7.4) | 2.3 (1.7-3.0) |
| No | 1.8 (1.5-2.1) | 0.8 (0.6-1.0) |
| *Used marijuana in past 12 months* | |  |
| Yes | 13.0 (9.0-18.5) | 7.0 (4.2-12.0) |
| No | 1.9 (1.7-2.2) | 0.7 (0.6-0.9) |

* All risk factors are significant at p<.001 using Pearson’s chi-squared test of independence.

** Other tobacco products include cigar, pipe, hookah, snus, smokeless tobacco, bidi, kretek, and dissolvable tobacco

| eTable 4A. Weighted association of ever e-cig use (wave 1) with subsequent past 12-month cigarette use (wave 2) among US youth in the PATH Study (N = 9,045), 4 Models with full regression results. | | | | | | | | | |
| --- | --- | --- | --- | --- | --- | --- | --- | --- | --- |
|  | **Reported Past 12-Month Cigarette Smoking in Wave 2** | | | | | | | | |
|  | **Model 1** | | **Model 2** | | **Model 3** | | **Model 4** | |  |
| **Wave 1 Variables** | **aOR (95% CI)** | **P-value** | **aOR (95% CI)** | **P-value** | **aOR (95% CI)** | **P-value** | **aOR (95% CI)** | **P-value** |  |
|  |  |  |  |  |  |  |  |  |  |
| Ever e-cigarette use | **5.55 (3.87-7.97)** | <.001 | **4.98 (3.38-7.34)** | <.001 | **3.34 (2.24-4.98)** | <.001 | **2.09 (1.26-3.48)** | .005 |  |
|  |  |  |  |  |  |  |  |  |  |
| **Exposure to Tobacco Users** |  |  |  |  |  |  |  |  |  |
| Family tobacco use |  |  | **1.39 (1.07-1.80)** | .015 | **1.38 (1.07-1.78)** | .01 | 1.33 (0.99-1.78) | .05 |  |
| Secondhand smoke |  |  | **1.66 (1.24-2.24)** | .001 | **1.41 (1.05-1.89)** | .02 | **1.48 (1.04-2.12)** | .03 |  |
|  |  |  |  |  |  |  |  |  |  |
| **Susceptibility** |  |  |  |  |  |  |  |  |  |
| Susceptible to cigarettes |  |  |  |  | **3.64 (2.73-4.85)** | <.001 | **3.13 (2.24-4.37)** | <.001 |  |
|  |  |  |  |  |  |  |  |  |  |
| **Behavioral Risk Factors** |  |  |  |  |  |  |  |  |  |
| Ever used other tobacco products* |  |  |  |  |  |  | 1.45 (0.89-2.34) | .13 |  |
| Used alcohol in past 12 months |  |  |  |  |  |  | **2.08 (1.39-3.10)** | .001 |  |
| Used marijuana in past 12 months |  |  |  |  |  |  | 2.03 (0.93-4.44) | .08 |  |
|  |  |  |  |  |  |  |  |  |  |
| **Sociodemographic Characteristics** |  |  |  |  |  |  |  |  |  |
| Female | **1.38 (1.08-1.75)** | .009 | **1.36 (1.33-2.15)** | <.001 | **1.31 (1.03-1.24)** | .03 | **1.40 (1.05-1.86)** | .02 |  |
| Age (REF: 12 - 14) |  |  |  |  |  |  |  |  |  |
| 15-17 | **1.73 (1.36-2.21)** | <.001 | **1.69 (1.33-2.15)** | <.001 | **1.58 (1.24-2.03)** | <.001 | **1.39 (1.04-1.87)** | .03 |  |
| Race/Ethnicity (REF: NH white) |  |  |  |  |  |  |  |  |  |
| NH black | **0.38 (0.24-0.59)** | <.001 | **0.38 (0.24-0.61)** | <.001 | **0.37 (0.23-0.60)** | <.001 | **0.38 (0.23-0.64)** | <.001 |  |
| Hispanic | **0.61 (0.45-0.83)** | .002 | **0.71 (0.51-0.98)** | .04 | **0.66 (0.47-0.92)** | .01 | **0.64 (0.44-0.93)** | .02 |  |
| NH other | **0.54 (0.32-0.90)** | .02 | **0.57 (0.34-0.95)** | .03 | **0.57 (0.33-0.96)** | .03 | **0.57 (0.34-0.96)** | .03 |  |
| Highest parental education (REF: High school/GED or less) | | | |  |  |  |  |  |  |
| Some college | 0.90 (0.70-1.17) | .44 | 0.93 (0.71-1.22) | .60 | 0.89 (0.68-1.17) | .40 | 1.02 (0.74-1.39) | .92 |  |
| College or higher | **0.60 (0.44-0.80)** | .001 | **0.71 (0.52-0.96)** | .03 | **0.66 (0.49-0.89)** | .01 | **0.60 (0.40-0.91)** | .02 |  |
| Grades > mostly B’s | **0.48 (0.38-0.59)** | <.001 | **0.53 (0.42-0.67)** | <.001 | **0.55 (0.44-0.69)** | <.001 | **0.56 (0.43-0.75)** | <.001 |  |

All bolded aORs are significant at p<.05.

All bolded aORs are significant at p<.05.

| eTable 4B. Weighted association of ever e-cig use (wave 2) with subsequent past 12-month cigarette use (wave 3) among US youth in the PATH Study (N = 8,668), 4 Models with full regression results. | | | | | | | | | |
| --- | --- | --- | --- | --- | --- | --- | --- | --- | --- |
|  | **Reported Past 12-Month Cigarette Smoking in Wave 3** | | | | | | | | |
|  | **Model 1** | | **Model 2** | | **Model 3** | | **Model 4** | |  |
| **Wave 2 Variables** | **aOR (95% CI)** | **P-value** | **aOR (95% CI)** | **P-value** | **aOR (95% CI)** | **P-value** | **aOR (95% CI)** | **P-value** |  |
|  |  |  |  |  |  |  |  |  |  |
| Ever e-cigarette use | **5.93 (4.07-8.63)** | <.001 | **3.61 (2.46-5.29)** | <.001 | **2.95 (2.03-4.28)** | <.001 | **2.10 (1.33-3.30)** | .002 |  |
|  |  |  |  |  |  |  |  |  |  |
| **Exposure to Tobacco Users** |  |  |  |  |  |  |  |  |  |
| Family tobacco use |  |  | 0.98 (0.72-1.33) | .88 | 1.01 (0.75-1.36) | .96 | 0.96 (0.70-1.31) | .78 |  |
| Secondhand smoke |  |  | **1.73 (1.26-2.39)** | .001 | **1.53 (1.10-2.12)** | .01 | **1.62 (1.15-2.29)** | .006 |  |
| Friends’ tobacco use |  |  | **2.85 (1.98-4.10)** | <.001 | **2.15 (1.47-3.14)** | <.001 | **1.74 (1.17-2.59)** | .007 |  |
|  |  |  |  |  |  |  |  |  |  |
| **Susceptibility** |  |  |  |  |  |  |  |  |  |
| Susceptible to cigarettes |  |  |  |  | **3.81 (2.60-5.58)** | <.001 | **3.55 (2.34-5.38)** | <.001 |  |
|  |  |  |  |  |  |  |  |  |  |
| **Behavioral Risk Factors** |  |  |  |  |  |  |  |  |  |
| Ever used other tobacco products* |  |  |  |  |  |  | 1.70 (0.92-3.16) | .09 |  |
| Used alcohol in past 12 months |  |  |  |  |  |  | 1.24 (0.87-1.77) | .23 |  |
| Used marijuana in past 12 months |  |  |  |  |  |  | **2.16 (1.11-4.18)** | .02 |  |
|  |  |  |  |  |  |  |  |  |  |
| **Sociodemographic Characteristics** |  |  |  |  |  |  |  |  |  |
| Female | 1.03 (0.79-1.34) | .83 | 1.02 (0.78-1.34) | .87 | 0.96 (0.74-1.25) | .74 | 0.94 (0.69-1.27) | .67 |  |
| Age (REF: 12 - 14) |  |  |  |  |  |  |  |  |  |
| 15-17 | **1.34 (1.03-1.75)** | .03 | 1.09 (0.80-1.47) | .59 | 1.15 (0.85-1.54) | .36 | 1.13 (0.80-1.61) | .48 |  |
| Race/Ethnicity (REF: NH white) |  |  |  |  |  |  |  |  |  |
| NH black | **0.29 (0.17-0.51)** | <.001 | **0.35 (0.19-0.63)** | .001 | **0.34 (0.18-0.62)** | .001 | **0.31 (0.14-0.64)** | .002 |  |
| Hispanic | **0.43 (0.29-0.65)** | <.001 | **0.53 (0.35-0.79)** | .002 | **0.47 (0.32-0.70)** | <.001 | **0.45 (0.28-0.72)** | .001 |  |
| NH other | **0.53 (0.31-0.91)** | .02 | **0.57 (0.33-0.99)** | .05 | 0.57 (0.33-1.00) | .05 | **0.40 (0.19-0.87)** | .02 |  |
| Highest parental education (REF: High school/GED or less) | | | |  |  |  |  |  |  |
| Some college | 0.89 (0.62-1.28) | .52 | 0.91 (0.64-1.29) | .60 | 0.82 (0.58-1.17) | .27 | 0.85 (0.58-1.25) | .41 |  |
| College or higher | 0.66 (0.38-1.15) | .14 | 0.74 (0.43-1.27) | .27 | 0.66 (0.38-1.15) | .14 | 0.58 (0.32-1.08) | .09 |  |
| Household income (REF: <50K) |  |  |  |  |  |  |  |  |  |
| 50k to 100k | 0.81 (0.59-1.10) | .18 | 0.91 (0.66-1.26) | .57 | 0.86 (0.61-1.19) | .36 | 0.79 (0.57-1.11) | .17 |  |
| > 100k | 0.81 (0.58-1.15) | .24 | 0.98 (0.68-1.42) | .93 | 0.90 (0.62-1.29) | .55 | 0.67 (0.42-1.08) | .10 |  |
| Grades > mostly B’s | **0.68 (0.49-0.96)** | .03 | 0.76 (0.54-1.07) | .11 | 0.81 (0.56-1.17) | .26 | 0.91 (0.59-1.39) | .64 |  |

| eTable 4C. Weighted association of ever e-cig use (wave 3) with subsequent past 12-month cigarette use (wave 4) among US youth in the PATH Study (N = 8,294), 4 Models with full regression results. | | | | | | | | | |
| --- | --- | --- | --- | --- | --- | --- | --- | --- | --- |
|  | **Reported Past 12-Month Cigarette Smoking in Wave 4** | | | | | | | | |
|  | **Model 1** | | **Model 2** | | **Model 3** | | **Model 4** | |  |
| **Wave 3 Variables** | **aOR (95% CI)** | **P-value** | **aOR (95% CI)** | **P-value** | **aOR (95% CI)** | **P-value** | **aOR (95% CI)** | **P-value** |  |
|  |  |  |  |  |  |  |  |  |  |
| Ever e-cigarette use | **5.53 (4.11-7.44)** | <.001 | **3.77 (2.80-5.07)** | <.001 | **3.16 (2.36-4.23)** | <.001 | **2.25 (1.55-3.27)** | <.001 |  |
|  |  |  |  |  |  |  |  |  |  |
| **Exposure to Tobacco Users** |  |  |  |  |  |  |  |  |  |
| Family tobacco use |  |  | **1.58 (1.11-2.25)** | .01 | **1.59 (1.10-2.28)** | .01 | **1.96 (1.32-2.91)** | .001 |  |
| Secondhand smoke |  |  | **1.84 (1.35-2.50)** | <.001 | **1.68 (1.23-2.30)** | .001 | 1.43 (0.98-2.08) | .07 |  |
| Friends’ tobacco use |  |  | **2.03 (1.61-2.58)** | <.001 | **1.62 (1.27-2.06)** | <.001 | 1.28 (0.96-1.71) | .09 |  |
|  |  |  |  |  |  |  |  |  |  |
| **Susceptibility** |  |  |  |  |  |  |  |  |  |
| Susceptible to cigarettes |  |  |  |  | **3.28 (2.50-4.32)** | <.001 | **3.26 (2.42-4.40)** | <.001 |  |
|  |  |  |  |  |  |  |  |  |  |
| **Behavioral Risk Factors** |  |  |  |  |  |  |  |  |  |
| Ever used other tobacco products* |  |  |  |  |  |  | 1.61 (0.86-2.98) | .13 |  |
| Used alcohol in past 12 months |  |  |  |  |  |  | **1.67 (1.16-2.39)** | .006 |  |
| Used marijuana in past 12 months |  |  |  |  |  |  | **2.27 (1.25-4.12)** | .007 |  |
|  |  |  |  |  |  |  |  |  |  |
| **Sociodemographic Characteristics** |  |  |  |  |  |  |  |  |  |
| Female | 0.90 (0.68-1.18) | .44 | 0.83 (0.63-1.10) | .20 | 0.79 (0.59-1.06) | .11 | 0.80 (0.59-1.09) | .15 |  |
| Age (REF: 12 - 14) |  |  |  |  |  |  |  |  |  |
| 15-17 | **1.91 (1.44-2.53)** | <.001 | **1.79 (1.35-2.38)** | <.001 | **1.86 (1.41-2.45)** | <.001 | **1.53 (1.10-2.13)** | .01 |  |
| Race/Ethnicity (REF: NH white) |  |  |  |  |  |  |  |  |  |
| NH black | **0.36 (0.21-0.63)** | <.001 | **0.46 (0.26-0.82)** | .009 | **0.45 (0.25-0.81)** | .008 | **0.48 (0.25-0.91)** | .03 |  |
| Hispanic | **0.60 (0.39-0.92)** | .02 | 0.75 (0.47-1.20) | .24 | 0.69 (0.42-1.11) | .13 | **0.55 (0.32-0.96)** | .03 |  |
| NH other | 0.62 (0.37-1.04) | .07 | 0.59 (0.34-1.05) | .07 | **0.56 (0.31-0.99)** | .05 | 0.62 (0.32-1.22) | .16 |  |
| Highest parental education (REF: High school/GED or less) | | | |  |  |  |  |  |  |
| Some college | 1.03 (0.73-1.46) | .86 | 1.01 (0.71-1.43) | .96 | 0.98 (0.68-1.41) | .89 | 0.88 (0.58-1.33) | .54 |  |
| College or higher | 0.94 (0.61-1.44) | .79 | 1.14 (0.76-1.72) | .53 | 1.03 (0.66-1.61) | .88 | 1.01 (0.63-1.62) | .95 |  |
| Household income (REF: <50K) |  |  |  |  |  |  |  |  |  |
| 50k to 100k | 1.01 (0.73-1.41) | .95 | 1.06 (0.75-1.48) | .75 | 1.05 (0.74-1.48) | .80 | 0.87 (0.56-1.33) | .51 |  |
| > 100k | 0.68 (0.43-1.06) | .09 | 0.80 (0.50-1.28) | .36 | 0.78 (0.48-1.27) | .32 | 0.63 (0.37-1.06) | .08 |  |
| Grades > mostly B’s | **0.57 (0.42-0.78)** | .001 | **0.65 (0.47-0.90)** | .01 | **0.65 (0.47-0.92)** | .01 | **0.61 (0.43-0.87)** | .006 |  |

All bolded aORs are significant at p<.05.

All bolded aORs are significant at p<.05.

| eTable 4D. Weighted association of ever e-cig use (wave 4) with subsequent past 12-month cigarette use (wave 4.5) among US youth in the PATH Study (N = 10,208), 4 Models with full regression results. | | | | | | | | | |
| --- | --- | --- | --- | --- | --- | --- | --- | --- | --- |
|  | **Reported Past 12-Month Cigarette Smoking in Wave 4.5** | | | | | | | | |
|  | **Model 1** | | **Model 2** | | **Model 3** | | **Model 4** | |  |
| **Wave 4 Variables** | **aOR (95% CI)** | **P-value** | **aOR (95% CI)** | **P-value** | **aOR (95% CI)** | **P-value** | **aOR (95% CI)** | **P-value** |  |
|  |  |  |  |  |  |  |  |  |  |
| Ever e-cigarette use | **4.96 (3.66-6.72)** | <.001 | **3.29 (2.38-4.56)** | <.001 | **2.71 (1.99-3.68)** | <.001 | 1.40 (0.91-2.14) | .12 |  |
|  |  |  |  |  |  |  |  |  |  |
| **Exposure to Tobacco Users** |  |  |  |  |  |  |  |  |  |
| Family tobacco use |  |  | **1.50 (1.10-2.04)** | .011 | **1.49 (1.10-2.03)** | .01 | **1.68 (1.18-2.40)** | .005 |  |
| Secondhand smoke |  |  | **1.53 (1.06-2.20)** | .02 | 1.38 (0.95-2.00) | .09 | 1.26 (0.83-1.93) | .28 |  |
| Friends’ tobacco use |  |  | **2.71 (2.04-3.60)** | <.001 | **2.22 (1.63-3.02)** | <.001 | **2.03 (1.42-2.89)** | <.001 |  |
|  |  |  |  |  |  |  |  |  |  |
| **Susceptibility** |  |  |  |  |  |  |  |  |  |
| Susceptible to cigarettes |  |  |  |  | **2.80 (1.98-3.95)** | <.001 | **2.44 (1.69-3.53)** | <.001 |  |
|  |  |  |  |  |  |  |  |  |  |
| **Behavioral Risk Factors** |  |  |  |  |  |  |  |  |  |
| Ever used other tobacco products* |  |  |  |  |  |  | **2.20 (1.27-3.82)** | .005 |  |
| Used alcohol in past 12 months |  |  |  |  |  |  | **1.49 (1.01-2.19)** | .04 |  |
| Used marijuana in past 12 months |  |  |  |  |  |  | **2.51 (1.34-4.71)** | .005 |  |
|  |  |  |  |  |  |  |  |  |  |
| **Sociodemographic Characteristics** |  |  |  |  |  |  |  |  |  |
| Female | 0.94 (0.72-1.22) | .63 | 0.87 (0.67-1.14) | .31 | 0.86 (0.65-1.12) | .25 | 0.78 (0.57-1.08) | .14 |  |
| Age (REF: 12 - 14) |  |  |  |  |  |  |  |  |  |
| 15-17 | **1.83 (1.35-2.49)** | <.001 | **1.63 (1.19-2.23)** | .003 | **1.70 (1.25-2.31)** | .001 | 1.39 (1.00-1.92) | .05 |  |
| Race/Ethnicity (REF: NH white) |  |  |  |  |  |  |  |  |  |
| NH black | **0.22 (0.11-0.45)** | <.001 | **0.27 (0.13-0.55)** | <.001 | **0.27 (0.13-0.55)** | <.001 | **0.25 (0.10-0.62)** | .003 |  |
| Hispanic | **0.68 (0.49-0.94)** | .02 | 0.87 (0.64-1.18) | .36 | 0.84 (0.61-1.14) | .26 | 0.81 (0.56-1.18) | .28 |  |
| NH other | 0.68 (0.44-1.05) | .09 | 0.73 (0.47-1.15) | .18 | 0.71 (0.45-1.12) | .14 | 0.67 (0.39-1.15) | .15 |  |
| Highest parental education (REF: High school/GED or less) | | | |  |  |  |  |  |  |
| Some college | 1.00 (0.68-1.46) | 1.0 | 1.03 (0.68-1.56) | .89 | 1.04 (0.69-1.56) | .86 | 1.01 (0.61-1.66) | .97 |  |
| College or higher | 1.01 (0.64-1.57) | 1.0 | 1.13 (0.71-1.79) | .60 | 1.07 (0.67-1.70) | .79 | 1.01 (0.60-1.72) | .97 |  |
| Household income (REF: <50K) |  |  |  |  |  |  |  |  |  |
| 50k to 100k | 1.01 (0.70-1.47) | .94 | 1.11 (0.75-1.65) | .60 | 1.13 (0.76-1.66) | .55 | 1.15 (0.76-1.74) | .52 |  |
| > 100k | 0.72 (0.47-1.10) | .13 | 0.83 (0.54-1.30) | .42 | 0.85 (0.55-1.32) | .47 | 0.94 (0.59-1.50) | .80 |  |
| Grades > mostly B’s | **0.43 (0.32-0.57)** | <.001 | **0.48 (0.35-0.65)** | <.001 | **0.48 (0.35-0.65)** | <.001 | **0.49 (0.33-0.72)** | <.001 |  |

All bolded aORs are significant at p<.05.

| eTable 4E. Weighted association of ever e-cig use (wave 4.5) with subsequent past 12-month cigarette use (wave 5) among US youth in the PATH Study (N = 11,560), 4 Models with full regression results. | | | | | | | | | |
| --- | --- | --- | --- | --- | --- | --- | --- | --- | --- |
|  | **Reported Past 12-Month Cigarette Smoking in Wave 5** | | | | | | | | |
|  | **Model 1** | | **Model 2** | | **Model 3** | | **Model 4** | |  |
| **Wave 4.5 Variables** | **aOR (95% CI)** | **P-value** | **aOR (95% CI)** | **P-value** | **aOR (95% CI)** | **P-value** | **aOR (95% CI)** | **P-value** |  |
|  |  |  |  |  |  |  |  |  |  |
| Ever e-cigarette use | **4.07 (2.86-5.81)** | <.001 | **2.28 (1.60-3.25)** | <.001 | **1.92 (1.34-2.75)** | <.001 | 1.35 (0.84-2.16) | .22 |  |
|  |  |  |  |  |  |  |  |  |  |
| **Exposure to Tobacco Users** |  |  |  |  |  |  |  |  |  |
| Family tobacco use |  |  | **1.65 (1.17-2.34)** | .005 | **1.65 (1.16-2.35)** | .006 | **1.68 (1.12-2.50)** | .01 |  |
| Secondhand smoke |  |  | **1.46 (1.01-2.12)** | .05 | 1.35 (0.93-1.94) | .11 | 1.08 (0.71-1.64) | .72 |  |
| Friends’ tobacco use |  |  | **2.84 (1.98-4.07)** | <.001 | **2.15 (1.49-3.11)** | <.001 | **2.03 (1.35-3.07)** | .001 |  |
|  |  |  |  |  |  |  |  |  |  |
| **Susceptibility** |  |  |  |  |  |  |  |  |  |
| Susceptible to cigarettes |  |  |  |  | **3.39 (2.49-4.61)** | <.001 | **2.95 (2.05-4.25)** | <.001 |  |
|  |  |  |  |  |  |  |  |  |  |
| **Behavioral Risk Factors** |  |  |  |  |  |  |  |  |  |
| Ever used other tobacco products* |  |  |  |  |  |  | **2.83 (1.54-5.21)** | .001 |  |
| Used alcohol in past 12 months |  |  |  |  |  |  | **1.85 (1.19-2.86)** | .006 |  |
| Used marijuana in past 12 months |  |  |  |  |  |  | 1.80 (0.94-3.46) | .08 |  |
|  |  |  |  |  |  |  |  |  |  |
| **Sociodemographic Characteristics** |  |  |  |  |  |  |  |  |  |
| Female | **1.00 (1.00-1.00)** | .05 | 1.00 (1.00-1.00) | .07 | 1.00 (1.00-1.00) | .11 | 1.00 (1.00-1.00) | .26 |  |
| Age (REF: 12 - 14) |  |  |  |  |  |  |  |  |  |
| 15-17 | **1.57 (1.12-2.18)** | .009 | **1.43 (1.02-2.00)** | .04 | **1.54 (1.11-2.14)** | .01 | 1.31 (0.90-1.89) | .15 |  |
| Race/Ethnicity (REF: NH white) |  |  |  |  |  |  |  |  |  |
| NH black | **0.37 (0.21-0.66)** | .001 | **0.40 (0.20-0.77)** | .006 | **0.41 (0.21-0.80)** | .01 | **0.45 (0.21-0.96)** | .04 |  |
| Hispanic | **0.63 (0.42-0.93)** | .02 | 0.86 (0.56-1.30) | .46 | 0.77 (0.50-1.19) | .24 | 0.69 (0.42-1.12) | .13 |  |
| NH other | 0.73 (0.44-1.22) | .23 | 0.79 (0.46-1.37) | .40 | 0.74 (0.42-1.31) | .30 | 0.84 (0.46-1.53) | .56 |  |
| Highest parental education (REF: High school/GED or less) | | | |  |  |  |  |  |  |
| Some college | 1.08 (0.69-1.68) | .74 | 1.08 (0.65-1.78) | .77 | 1.08 (0.66-1.76) | .76 | 1.06 (0.60-1.85) | .85 |  |
| College or higher | 0.67 (0.40-1.14) | .14 | 0.78 (0.45-1.37) | .39 | 0.74 (0.42-1.30) | .29 | 0.66 (0.35-1.23) | .18 |  |
| Household income (REF: <50K) |  |  |  |  |  |  |  |  |  |
| 50k to 100k | 0.86 (0.59-1.26) | .44 | 0.84 (0.57-1.24) | .37 | 0.85 (0.57-1.26) | .41 | 0.63 (0.38-1.04) | .07 |  |
| > 100k | 0.58 (0.31-1.09) | .09 | 0.65 (0.34-1.27) | .20 | 0.65 (0.33-1.27) | .20 | 0.61 (0.28-1.34) | .22 |  |
| Grades > mostly B’s | **0.64 (0.47-0.88)** | .006 | **0.66 (0.48-0.91)** | .01 | **0.66 (0.47-0.93)** | .02 | **0.64 (0.43-0.95)** | .03 |  |

All bolded aORs are significant at p<.05.

| eTable 5A. Weighted association of ever e-cig use (wave 1) with subsequent past 30-day cigarette use (wave 2) among US youth in the PATH Study (N = 9,045), 4 Models with full regression results. | | | | | | | | |
| --- | --- | --- | --- | --- | --- | --- | --- | --- |
|  | **Reported Past 30-Day Cigarette Smoking in Wave 2** | | | | | | | |
|  | **Model 1** | | **Model 2** | | **Model 3** | | **Model 4** | |
| **Wave 1 Variables** | **aOR (95% CI)** | **P-value** | **aOR (95% CI)** | **P-value** | **aOR (95% CI)** | **P-value** | **aOR (95% CI)** | **P-value** |
|  |  |  |  |  |  |  |  |  |
| Ever e-cigarette use | **4.32 (2.42-7.70)** | <.001 | **3.74 (2.04-6.84)** | <.001 | **2.45 (1.36-4.42)** | .003 | 1.41 (0.64-3.09) | .39 |
|  |  |  |  |  |  |  |  |  |
| **Exposure to Tobacco Users** |  |  |  |  |  |  |  |  |
| Family tobacco use |  |  | **1.53 (1.01-2.33)** | .05 | **1.53 (1.01-2.34)** | .05 | 1.30 (0.80-2.13) | .29 |
| Secondhand smoke |  |  | **1.94 (1.33-2.81)** | .001 | **1.61 (1.11-2.33)** | .01 | 1.56 (0.96-2.52) | .07 |
|  |  |  |  |  |  |  |  |  |
| **Susceptibility** |  |  |  |  |  |  |  |  |
| Susceptible to cigarettes |  |  |  |  | **4.04 (2.64-6.20)** | <.001 | **3.38 (2.09-5.45)** | <.001 |
|  |  |  |  |  |  |  |  |  |
| **Behavioral Risk Factors** |  |  |  |  |  |  |  |  |
| Ever used other tobacco products* |  |  |  |  |  |  | 1.58 (0.82-3.05) | .17 |
| Used alcohol in past 12 months |  |  |  |  |  |  | 1.46 (0.79-2.69) | .23 |
| Used marijuana in past 12 months |  |  |  |  |  |  | 2.12 (0.75-6.03) | .16 |
|  |  |  |  |  |  |  |  |  |
| **Sociodemographic Characteristics** | |  |  |  |  |  |  |  |
| Female | 1.27 (0.89-1.83) | .19 | 1.22 (0.85-1.74) | .27 | 1.16 (0.81-1.67) | .41 | 1.36 (0.89-2.07) | .16 |
| Age (REF: 12 - 14) |  |  |  |  |  |  |  |  |
| 15-17 | **2.15 (1.45-3.20)** | <.001 | **2.05 (1.39-3.03)** | <.001 | **1.92 (1.30-2.86)** | .001 | **1.72 (1.06-2.79)** | .03 |
| Race/Ethnicity (REF: NH white) |  |  |  |  |  |  |  |  |
| NH black | **0.31 (0.16-0.61)** | .001 | **0.33 (0.17-0.66)** | .002 | **0.33 (0.17-0.64)** | .001 | **0.39 (0.19-0.78)** | .008 |
| Hispanic | **0.45 (0.28-0.73)** | .002 | **0.56 (0.34-0.94)** | .03 | **0.52 (0.31-0.88)** | .02 | **0.54 (0.30-0.98)** | .04 |
| NH other | 0.48 (0.23-1.02) | .06 | 0.52 (0.24-1.10) | .09 | 0.52 (0.24-1.12) | .10 | 0.48 (0.22-1.05) | .07 |
| Highest parental education (REF: High school/GED or less) | | |  |  |  |  |  |  |
| Some college | 0.85 (0.53-1.35) | .49 | 0.91 (0.57-1.46) | .70 | 0.87 (0.54-1.39) | .55 | 1.02 (0.60-1.76) | .93 |
| College or higher | **0.38 (0.21-0.66)** | .001 | **0.48 (0.27-0.85)** | .01 | **0.44 (0.25-0.78)** | .005 | **0.38 (0.18-0.81)** | .01 |
| Grades > mostly B’s | **0.46 (0.33-0.64)** | <.001 | **0.55 (0.40-0.76)** | <.001 | **0.58 (0.42-0.81)** | .002 | **0.55 (0.38-0.79)** | .002 |

| eTable 5B. Weighted association of ever e-cig use (wave 2) with subsequent past 30-day cigarette use (wave 3) among US youth in the PATH Study (N = 8,668), 4 Models with full regression results. | | | | | | | | |
| --- | --- | --- | --- | --- | --- | --- | --- | --- |
|  | **Reported Past 30-Day Cigarette Smoking in Wave 3** | | | | | | | |
|  | **Model 1** | | **Model 2** | | **Model 3** | | **Model 4** | |
| **Wave 2 Variables** | **aOR (95% CI)** | **P-value** | **aOR (95% CI)** | **P-value** | **aOR (95% CI)** | **P-value** | **aOR (95% CI)** | **P-value** |
|  |  |  |  |  |  |  |  |  |
| Ever e-cigarette use | **5.71 (3.11-10.49)** | <.001 | **3.36 (1.84-6.13)** | <.001 | **2.80 (1.58-4.96)** | .001 | 1.41 (0.67-2.98) | .37 |
|  |  |  |  |  |  |  |  |  |
| **Exposure to Tobacco Users** |  |  |  |  |  |  |  |  |
| Family tobacco use |  |  | 1.05 (0.59-1.86) | .87 | 1.09 (0.62-1.92) | .76 | 0.98 (0.63-1.53) | .93 |
| Secondhand smoke |  |  | **2.37 (1.30-4.33)** | .005 | **2.15 (1.17-3.95)** | .01 | **2.54 (1.29-5.02)** | .008 |
| Friends’ tobacco use |  |  | **2.81 (1.48-5.35)** | .002 | **2.16 (1.09-4.29)** | .03 | 1.94 (0.90-4.17) | .09 |
|  |  |  |  |  |  |  |  |  |
| **Susceptibility** |  |  |  |  |  |  |  |  |
| Susceptible to cigarettes |  |  |  |  | **2.94 (1.53-5.64)** | .001 | **2.65 (1.26-5.55)** | .01 |
|  |  |  |  |  |  |  |  |  |
| **Behavioral Risk Factors** |  |  |  |  |  |  |  |  |
| Ever used other tobacco products* | |  |  |  |  |  | 2.60 (1.00-6.73) | .05 |
| Used alcohol in past 12 months |  |  |  |  |  |  | 1.06 (0.58-1.96) | .84 |
| Used marijuana in past 12 months | |  |  |  |  |  | **2.81 (1.02-7.72)** | .05 |
|  |  |  |  |  |  |  |  |  |
| **Sociodemographic Characteristics** | |  |  |  |  |  |  |  |
| Female | **1.73 (1.10-2.70)** | .02 | **1.66 (1.04-2.65)** | .03 | 1.56 (0.98-2.48) | .06 | **1.88 (1.10-3.23)** | .02 |
| Age (REF: 12 - 14) |  |  |  |  |  |  |  |  |
| 15-17 | 1.51 (0.90-2.51) | .12 | 1.37 (0.73-2.56) | .32 | 1.44 (0.77-2.72) | .25 | 1.34 (0.62-2.90) | .45 |
| Race/Ethnicity (REF: NH white) |  |  |  |  |  |  |  |  |
| NH black | **0.19 (0.07-0.53)** | .002 | **0.26 (0.10-0.73)** | .01 | **0.26 (0.09-0.71)** | .01 | **0.26 (0.08-0.86)** | .03 |
| Hispanic | **0.35 (0.18-0.66)** | .002 | **0.49 (0.27-0.92)** | .03 | **0.45 (0.25-0.82)** | .01 | **0.42 (0.20-0.86)** | .02 |
| NH other | 0.63 (0.24-1.65) | .35 | 0.70 (0.27-1.83) | .46 | 0.71 (0.27-1.83) | .48 | 0.36 (0.08-1.55) | .17 |
| Highest parental education (REF: High school/GED or less) | | |  |  |  |  |  |  |
| Some college | 0.92 (0.45-1.86) | .81 | 1.02 (0.51-2.07) | .95 | 0.94 (0.47-1.87) | .86 | 0.85 (0.39-1.83) | .67 |
| College or higher | 0.87 (0.37-2.01) | .74 | 1.10 (0.48-2.50) | .82 | 1.00 (0.45-2.25) | .99 | 0.90 (0.39-2.10) | .81 |
| Household income (REF: <50K) |  |  |  |  |  |  |  |  |
| 50k to 100k | 0.55 (0.30-1.04) | .06 | 0.67 (0.35-1.29) | .23 | 0.64 (0.33-1.24) | .19 | 0.63 (0.29-1.35) | .23 |
| > 100k | 0.55 (0.28-1.07) | .08 | 0.73 (0.36-1.48) | .38 | 0.67 (0.33-1.36) | .26 | 0.48 (0.21-1.10) | .08 |
| Grades > mostly B’s | **0.51 (0.29-0.89)** | .02 | **0.55 (0.32-0.95)** | .03 | 0.58 (0.34-1.01) | .06 | 0.63 (0.31-1.29) | .20 |

All bolded aORs are significant at p<.05.

| eTable 5C. Weighted association of ever e-cig use (wave 3) with subsequent past 30-day cigarette use (wave 4) among US youth in the PATH Study (N = 8,294), 4 Models with full regression results. | | | | | | | | |
| --- | --- | --- | --- | --- | --- | --- | --- | --- |
|  | **Reported Past 30-Day Cigarette Smoking in Wave 4** | | | | | | | |
|  | **Model 1** | | **Model 2** | | **Model 3** | | **Model 4** | |
| **Wave 3 Variables** | **aOR (95% CI)** | **P-value** | **aOR (95% CI)** | **P-value** | **aOR (95% CI)** | **P-value** | **aOR (95% CI)** | **P-value** |
|  |  |  |  |  |  |  |  |  |
| Ever e-cigarette use | **6.04 (3.89-9.36)** | <.001 | **3.72 (2.43-5.68)** | <.001 | **3.04 (2.00-4.63)** | <.001 | **2.16 (1.18-3.97)** | .01 |
|  |  |  |  |  |  |  |  |  |
| **Exposure to Tobacco Users** |  |  |  |  |  |  |  |  |
| Family tobacco use |  |  | 1.57 (0.96-2.56) | .07 | 1.56 (0.94-2.60) | .09 | **1.97 (1.12-3.46)** | .02 |
| Secondhand smoke |  |  | **1.86 (1.17-2.96)** | .01 | **1.70 (1.05-2.76)** | .03 | 1.52 (0.92-2.50) | .10 |
| Friends’ tobacco use |  |  | **2.32 (1.41-3.81)** | .001 | **1.83 (1.12-3.00)** | .02 | 1.34 (0.76-2.37) | .31 |
|  |  |  |  |  |  |  |  |  |
| **Susceptibility** |  |  |  |  |  |  |  |  |
| Susceptible to cigarettes |  |  |  |  | **3.56 (2.18-5.82)** | <.001 | **3.64 (2.08-6.37)** | <.001 |
|  |  |  |  |  |  |  |  |  |
| **Behavioral Risk Factors** |  |  |  |  |  |  |  |  |
| Ever used other tobacco products* | |  |  |  |  |  | 1.15 (0.49-2.68) | .74 |
| Used alcohol in past 12 months |  |  |  |  |  |  | 1.53 (0.88-2.63) | .13 |
| Used marijuana in past 12 months | |  |  |  |  |  | **2.76 (1.27-6.03)** | .01 |
|  |  |  |  |  |  |  |  |  |
| **Sociodemographic Characteristics** | |  |  |  |  |  |  |  |
| Female | 0.95 (0.63-1.42) | .79 | 0.82 (0.55-1.24) | .34 | 0.78 (0.51-1.18) | .24 | 0.79 (0.49-1.26) | .31 |
| Age (REF: 12 - 14) |  |  |  |  |  |  |  |  |
| 15-17 | **2.17 (1.40-3.38)** | .001 | **2.22 (1.39-3.54)** | .001 | **2.30 (1.46-3.64)** | <.001 | **2.16 (1.31-3.54)** | .003 |
| Race/Ethnicity (REF: NH white) |  |  |  |  |  |  |  |  |
| NH black | **0.28 (0.12-0.68)** | .005 | **0.37 (0.15-0.91)** | .03 | **0.36 (0.15-0.90)** | .03 | **0.36 (0.13-0.97)** | .04 |
| Hispanic | **0.48 (0.27-0.83)** | .01 | 0.58 (0.31-1.09) | .09 | 0.52 (0.27-1.01) | .05 | **0.35 (0.15-0.83)** | .02 |
| NH other | 0.49 (0.24-1.01) | .05 | 0.50 (0.22-1.10) | .09 | 0.46 (0.20-1.06) | .07 | **0.51 (1.31-3.54)** | .003 |
| Highest parental education (REF: High school/GED or less) | | |  |  |  |  |  |  |
| Some college | 1.31 (0.79-2.17) | .30 | 1.22 (0.74-2.01) | .43 | 1.18 (0.70-2.01) | .53 | 1.03 (0.58-1.83) | .92 |
| College or higher | 1.10 (0.65-1.84) | .73 | 1.22 (0.73-2.02) | .45 | 1.09 (0.64-1.87) | .74 | 1.08 (0.59-1.99) | .80 |
| Household income (REF: <50K) |  |  |  |  |  |  |  |  |
| 50k to 100k | 0.79 (0.50-1.27) | .33 | 0.88 (0.54-1.45) | .62 | 0.86 (0.52-1.42) | .55 | 0.78 (0.44-1.37) | .38 |
| > 100k | **0.46 (0.26-0.82)** | .009 | 0.56 (0.30-1.06) | .07 | 0.54 (0.29-1.02) | .06 | **0.41 (0.20-0.83)** | .01 |
| Grades > mostly B’s | **0.54 (0.36-0.80)** | .003 | **0.62 (0.40-0.95)** | .03 | **0.63 (0.41-0.97)** | .03 | **0.61 (0.37-0.99)** | .05 |

All bolded aORs are significant at p<.05.

All bolded aORs are significant at p<.05.

| eTable 5D. Weighted association of ever e-cig use (wave 4) with subsequent past 30-day cigarette use (wave 4.5) among US youth in the PATH Study (N = 10,208), 4 Models with full regression results. | | | | | | | | |
| --- | --- | --- | --- | --- | --- | --- | --- | --- |
|  | **Reported Past 30-Day Cigarette Smoking in Wave 4.5** | | | | | | | |
|  | **Model 1** | | **Model 2** | | **Model 3** | | **Model 4** | |
| **Wave 4 Variables** | **aOR (95% CI)** | **P-value** | **aOR (95% CI)** | **P-value** | **aOR (95% CI)** | **P-value** | **aOR (95% CI)** | **P-value** |
|  |  |  |  |  |  |  |  |  |
| Ever e-cigarette use | **4.14 (2.71-6.32)** | <.001 | **2.71 (1.72-4.26)** | <.001 | **2.30 (1.45-3.66)** | .001 | 1.11 (0.57-2.16) | .75 |
|  |  |  |  |  |  |  |  |  |
| **Exposure to Tobacco Users** |  |  |  |  |  |  |  |  |
| Family tobacco use |  |  | **2.14 (1.31-3.51)** | .003 | **2.16 (1.32-3.54)** | .002 | **2.39 (1.31-4.34)** | .005 |
| Secondhand smoke |  |  | 1.33 (0.77-2.29) | .30 | 1.20 (0.69-2.09) | .52 | 1.06 (0.55-2.07) | .86 |
| Friends’ tobacco use |  |  | **3.10 (1.93-4.97)** | <.001 | **2.61 (1.58-4.30)** | <.001 | **2.37 (1.27-4.39)** | .007 |
|  |  |  |  |  |  |  |  |  |
| **Susceptibility** |  |  |  |  |  |  |  |  |
| Susceptible to cigarettes |  |  |  |  | **2.46 (1.46-4.13)** | .001 | **2.70 (1.40-5.18)** | .003 |
|  |  |  |  |  |  |  |  |  |
| **Behavioral Risk Factors** |  |  |  |  |  |  |  |  |
| Ever used other tobacco products* |  |  |  |  |  |  | 2.07 (0.89-4.80) | .09 |
| Used alcohol in past 12 months |  |  |  |  |  |  | 0.94 (0.51-1.72) | .85 |
| Used marijuana in past 12 months |  |  |  |  |  |  | **4.07 (1.87-8.84)** | .001 |
|  |  |  |  |  |  |  |  |  |
| **Sociodemographic Characteristics** | |  |  |  |  |  |  |  |
| Female | 1.13 (0.71-1.81) | .60 | 1.07 (0.65-1.76) | .80 | 1.05 (0.63-1.73) | .85 | 1.00 (0.59-1.69) | .99 |
| Age (REF: 12 - 14) |  |  |  |  |  |  |  |  |
| 15-17 | **2.15 (1.24-3.72)** | .007 | **2.02 (1.15-3.56)** | .02 | **2.11 (1.20-3.70)** | .01 | 1.68 (0.91-3.11) | .10 |
| Race/Ethnicity (REF: NH white) |  |  |  |  |  |  |  |  |
| NH black | **0.07 (0.02-0.33)** | .001 | **0.09 (0.02-0.45)** | .003 | **0.10 (0.02-0.46)** | .004 | **0.12 (0.03-0.60)** | .01 |
| Hispanic | **0.46 (0.25-0.86)** | .02 | 0.63 (0.33-1.20) | .16 | 0.61 (0.32-1.19) | .14 | 0.59 (0.26-1.38) | .22 |
| NH other | **0.38 (0.16-0.92)** | .03 | 0.41 (0.16-1.03) | .06 | 0.40 (0.16-1.02) | .06 | 0.36 (0.13-1.01) | .05 |
| Highest parental education (REF: High school/GED or less) | | |  |  |  |  |  |  |
| Some college | 1.33 (0.82-2.18) | .25 | 1.48 (0.84-2.59) | .17 | 1.49 (0.86-2.59) | .15 | 1.46 (0.74-2.89) | .28 |
| College or higher | 1.35 (0.69-2.62) | .38 | 1.67 (0.86-3.24) | .13 | 1.59 (0.81-3.12) | .18 | 1.29 (0.56-2.93) | .55 |
| Household income (REF: <50K) |  |  |  |  |  |  |  |  |
| 50k to 100k | 0.73 (0.46-1.17) | .19 | 0.81 (0.50-1.31) | .39 | 0.83 (0.52-1.34) | .45 | 0.98 (0.57-1.68) | .93 |
| > 100k | **0.44 (0.22-0.91)** | .03 | 0.55 (0.27-1.12) | .10 | 0.56 (0.28-1.15) | .11 | 0.67 (0.31-1.48) | .32 |
| Grades > mostly B’s | **0.25 (0.16-0.40)** | <.001 | **0.27 (0.16-0.45)** | <.001 | **0.27 (0.16-0.46)** | <.001 | **0.34 (0.18-0.62)** | <.001 |

All bolded aORs are significant at p<.05.

| eTable 5E. Weighted association of ever e-cig use (wave 4.5) with subsequent past 30-day cigarette use (wave 5) among US youth in the PATH Study (N = 11,560), 4 Models with full regression results. | | | | | | | | |
| --- | --- | --- | --- | --- | --- | --- | --- | --- |
|  | **Reported Past 30-Day Cigarette Smoking in Wave 5** | | | | | | | |
|  | **Model 1** | | **Model 2** | | **Model 3** | | **Model 4** | |
| **Wave 4.5 Variables** | **aOR (95% CI)** | **P-value** | **aOR (95% CI)** | **P-value** | **aOR (95% CI)** | **P-value** | **aOR (95% CI)** | **P-value** |
|  |  |  |  |  |  |  |  |  |
| Ever e-cigarette use | **3.26 (1.81-5.86)** | <.001 | **1.78 (1.01-3.11)** | .05 | 1.53 (0.87-2.69) | .14 | 1.21 (0.59-2.48) | .60 |
|  |  |  |  |  |  |  |  |  |
| **Exposure to Tobacco Users** |  |  |  |  |  |  |  |  |
| Family tobacco use |  |  | **2.13 (1.23-3.69)** | .008 | **2.11(1.22-3.67)** | .008 | **2.13 (1.18-3.86)** | .01 |
| Secondhand smoke |  |  | 1.33 (0.72-2.45) | .36 | 1.24 (0.67-2.28) | .49 | 1.10 (0.54-2.24) | .79 |
| Friends’ tobacco use |  |  | **2.93 (1.57-5.45)** | .001 | **2.26 (1.21-4.24)** | .01 | **2.23 (1.18-4.23)** | .01 |
|  |  |  |  |  |  |  |  |  |
| **Susceptibility** |  |  |  |  |  |  |  |  |
| Susceptible to cigarettes |  |  |  |  | **2.96 (1.78-4.94)** | <.001 | **2.66 (1.50-4.74)** | .001 |
|  |  |  |  |  |  |  |  |  |
| **Behavioral Risk Factors** |  |  |  |  |  |  |  |  |
| Ever used other tobacco products* |  |  |  |  |  |  | **2.77 (1.16-6.58)** | .02 |
| Used alcohol in past 12 months |  |  |  |  |  |  | **2.16 (1.13-4.11)** | .02 |
| Used marijuana in past 12 months |  |  |  |  |  |  | 1.90 (0.74-4.89) | .18 |
|  |  |  |  |  |  |  |  |  |
| **Sociodemographic Characteristics** | |  |  |  |  |  |  |  |
| Female | **1.00 (1.00-1.00)** | .04 | **1.00 (1.00-1.00)** | .04 | 1.00 (1.00-1.00) | .06 | 1.00 (1.00-1.00) | .38 |
| Age (REF: 12 - 14) |  |  |  |  |  |  |  |  |
| 15-17 | **1.78 (1.02-3.11)** | .04 | 1.57 (0.89-2.77) | .12 | 1.67 (0.97-2.90) | .07 | 1.51 (0.82-2.79) | .18 |
| Race/Ethnicity (REF: NH white) |  |  |  |  |  |  |  |  |
| NH black | **0.23 (0.06-0.89)** | .03 | 0.31 (0.08-1.23) | .10 | 0.32 (0.08-1.31) | .11 | 0.46 (0.11-1.85) | .27 |
| Hispanic | 0.57 (0.30-1.08) | .08 | 0.80 (0.41-1.54) | .50 | 0.74 (0.37-1.46) | .38 | 0.74 (0.34-1.64) | .46 |
| NH other | 0.63 (0.20-2.00) | .43 | 0.69 (0.21-2.26) | .54 | 0.65 (0.19-2.19) | .48 | 0.67 (0.16-2.80) | .58 |
| Highest parental education (REF: High school/GED or less) | | |  |  |  |  |  |  |
| Some college | 1.27 (0.62-2.61) | .51 | 1.20 (0.56-2.58) | .63 | 1.20 (0.56-2.58) | .64 | 1.18 (0.55-2.54) | .67 |
| College or higher | 0.86 (0.38-1.93) | .71 | 0.97 (0.43-2.20) | .94 | 0.93 (0.41-2.14) | .87 | 0.76 (0.33-1.75) | .51 |
| Household income (REF: <50K) |  |  |  |  |  |  |  |  |
| 50k to 100k | 0.67 (0.39-1.14) | .14 | 0.72 (0.42-1.22) | .22 | 0.73 (0.42-1.25) | .25 | 0.51 (0.25-1.05) | .07 |
| > 100k | 0.50 (0.23-1.07) | .08 | 0.60 (0.25-1.44) | .25 | 0.60 (0.24-1.46) | .26 | 0.61 (0.23-1.62) | .32 |
| Grades > mostly B’s | **0.59 (0.39-0.89)** | .01 | 0.68 (0.44-1.04) | .08 | 0.69 (0.44-1.08) | .10 | 0.60 (0.34-1.03) | .06 |

eTable 6. Limiting sample size* of outcome variables (past 12-month and past 30-day cigarette use) without survey weights.

|  | **Limiting Sample Size** | |
| --- | --- | --- |
| **Waves** | Past 12-Month Use | Past 30-Day Use |
| Waves 1-2 | 334 | 140 |
| Waves 2-3 | 272 | 105 |
| Waves 3-4 | 278 | 123 |
| Waves 4-4.5 | 273 | 109 |
| Waves 4.5-5 | 257 | 90 |

Notes.

* Limiting sample size is the number of occurrences of the less common event, aka number (unweighted) of baseline never cigarette smokers who reported past 12-month or past 30-day cigarette smoking in the subsequent wave.
